# “HerdGPS-Preprocessor”—A Tool to Preprocess Herd Animal GPS Data; Applied to Evaluate Contact Structures in Loose-Housing Horses

**DOI:** 10.3390/ani10101932

**Published:** 2020-10-21

**Authors:** Jennifer Salau, Frederik Hildebrandt, Irena Czycholl, Joachim Krieter

**Affiliations:** Institute of Animal Breeding and Husbandry, Christian-Albrechts-University Kiel, Olshausenstraße 40, 24098 Kiel, Germany; fhildebrandt@tierzucht.uni-kiel.de (F.H.); iczycholl@tierzucht.uni-kiel.de (I.C.); jkrieter@tierzucht.uni-kiel.de (J.K.)

**Keywords:** GPS data, data preprocessing, precision livestock farming, herd structure, network analysis, animal tracking

## Abstract

**Simple Summary:**

Many studies are working with GPS sensors to track farm animals in order to gather information related to the animals’ health, the use of space and resources provided to the animals or their social contacts. When a herd of animals is tracked uninterruptedly, a huge amount of data is generated. Furthermore, problems like the limited battery life of wireless sensors have to be dealt with. This article presents the software “HerdGPS-Preprocessor” for the preparation of GPS data collected from an animal herd. The GPS data is provided cleaned and organized, such that the output files enable direct statistical analysis. Additionally, contact lists are output to enable network analyses. This study also delivers an example analysis with the “HerdGPS-Preprocessor”, in which GPS data of forty horses kept in a loose-housing barn were used to visualise and analyse contact networks. From the calculation of network parameters (density, diameter) and metrics associated to the clique structure of the horses, resting and activity phases could be distinguished. Furthermore, by network analysis it was shown that pasture opening and the visiting times of horse owners affect the contact structure. Thus, “HerdGPS-Preprocessor” is a valuable tool in studies collecting GPS data from herd animals.

**Abstract:**

Sensors delivering information on the position of farm animals have been widely used in precision livestock farming. Global Positioning System (GPS) sensors are already known from applications in military, private and commercial environments, and their application in animal science is increasing. However, as trade-offs between sensor cost, battery life and sensor weight have to be made, GPS based studies scheduling long data collection periods and including a high number of animals, have to deal with problems like high hardware costs and data disruption during recharging of sensors. Furthermore, human–animal interaction due to sensor changing at the end of battery life interferes with the animal behaviour under analysis. The present study thus proposes a setting to deal with these challenges and offers the software tool “HerdGPS-Preprocessor”, because collecting position data from multiple animals nonstop for several weeks produces a high amount of raw data which needs to be sorted, preprocessed and provided in a suitable format per animal and day. The software tool “HerdGPS-Preprocessor” additionally outputs contact lists to enable a straight analysis of animal contacts. The software tool was exemplarily deployed for one month of daily and continuous GPS data of 40 horses in a loose-housing boarding facility in northern Germany. Contact lists were used to generate separate networks for every hour, which are then analysed with regard to the network parameter density, diameter and clique structure. Differences depending on the day and the day time could be observed. More dense networks with more and larger cliques were determined in the hours prior to the opening of additional pasture.

## 1. Introduction

A topic widely dealt with in the field of precision livestock farming is the analysis of position data gathered from farm animals. Real-time location systems based on radio frequency identification (RFID) [1] or ultra wide band (UWB) [2,3] or radar based systems [4] were applied in order to evaluate animal positions indoors, to contribute to herd management as well as to analyse herd structure and social behaviour in barn environment [5]. Proximity loggers determine the distance between animals, and are a way to define contacts between animals without specifying the animal position in advance. These sensors were used in [6] to describe the social structure of a lactating cattle on pasture and found that animals formed differentiated relationships with positive assortment by traits as lactation number, breed or milk production. Reference [7] used proximity loggers to determine bull-cow in comparison between cows were in estrous and not in estrous. In pasture based animal farming, activity logger [8,9,10] and sensors using the Global Positioning System (GPS) [11] came to use.

GPS sensors are a very widespread tool in military and commercial settings, or in every day use to track cars, domestic animals or persons. In the past years, additionally, their applications in science are increasing. Without any claim to completeness transport policy [12], space research [13,14], social sciences [15], geosciences [16,17] and animal sciences can be named. Exemplarily, reference [18] tracked 180 cattle using GPS sensors to monitor spatial impact of grazing on rangelands and to adjust stocking rates. In [19] a kit to monitor herd activity and cattle behaviour was proposed and individual daily travel distances were determined. Reference [20] used GPS sensors to analyse cattle behaviour. With Foraging, Lying, Standing and Walking, four types of behaviour could be found in the GPS data by training classification and regression trees on the pattern of the characteristic movement metrics of these behaviours. Valuable insights could be gathered from hard data concerning the distances an animal has walked per day or the speed with which an animal moves about potential lameness [21] or with regard to oestrus detection [3,22]. Important work has been carried out on the walked distances of feral horses, stating averaged distances varying from 3.5 km per day [23] to 9 km per day [24] to 15.9 km per day [25]. In [26] it was distinguished between Konik horses’ walking distances in the forest (6.5 km per day) and the meadow (2.6 km per day). With [27] a recently published study reports the walked daily distances of a large, inhomogeneous group of domestic horses to be 8.43 km. From position data also the evaluation of the animals’ space utilization becomes possible and promises important scientific findings towards animal husbandry and health [28], and the use of provided resources [29]. Furthermore, contacts between animals as well as information on the usage of resources like feed, water, or lying areas can be analysed from position data using the methods of network analysis. Network analysis [30,31] has become a valuable instrument in animal sciences as it provides meaningful parameters to describe and research social structures [6,32,33], animal behaviour [11,34] or interactions [35,36,37,38] and disease spreading [39,40,41,42,43]).

Using GPS sensors, position information of various animals could be gathered without visually inspecting multiple hours of recordings from video surveillance cameras [44,45,46] or developing automated image processing tools [47,48,49,50]. As an additional advantage, GPS sensors are easy to attach without disturbing the animals in their every day activities. Drawbacks are an effect of the sampling interval –for example, on the individually walked distance [19]– and the limited battery runtime of the sensors. Products providing longer battery life, often are more expensive which could lead to high costs when a large number of animals needs to be equipped. Larger batteries also make the sensors heavier in weight and prone to disturb the animals. In contrast to using GPS sensors only for limited specified periods—for example, during horse training sessions [51,52]—the sensors need to be recharged in regular time intervals, when GPS data is collected for a longer time period. This leads either to gaps in data collection or the necessity to regularly change the battery for all animals participating in the study.

The present article provides a possibility for a low cost setting for GPS based studies over long periods and with a high number of animals in which disruptions in the data and the interaction with the animals under analysis are kept to a minimum. The exemplary object of investigation was a herd of horses in a loose-housing boarding facility in Schleswig-Holstein, Northern Germany. Two sensors attached to collars were associated to each horse, and in order to minimise the time the horses were disturbed, at the end of battery life a quick exchange of collars was performed. Nonstop recording the positions of multiple animals over a lengthy period of time raised a high amount of raw data which needed to be filtered, preprocessed and provided in a suitable format per animal and day. Furthermore, it seemed sensible to draw information on contacts between the animals during preprocessing the positional data, in order to spare computational time and to offer the opportunity for an immediate analysis of contact networks. Therefore, in this article a software tool “HerdGPS-Preprocessor” for GPS data recorded from a group of herd animals is proposed, that delivers filtered and preprocessed GPS data of individual animals as well as contact lists to document their social interaction. With this, network analysis or inferential statistics with regard to various scientific problems become possible. The benefits of “HerdGPS-Preprocessor” were illustrated using one months GPS data of forty horses.

## 2. Materials and Methods

### 2.1. Data Collection

In a loose-housing boarding facility in Schleswig-Holstein, Northern Germany a herd of horses was equipped with Qstarz BT-Q1000XT GPS sensors. The sensors were attached to collars and covered with duct tape to prevent the horses from removing each others sensors. The collars were designed to be worn by horses full time around the upper part of the neck without disturbing the animals during feeding, moving, social interactions or lying down. In the boarding facility under analysis, the horses could move around freely and had free access to water, straw and lying areas. Hay and concentrated feed could be retrieved from automated feeding dispensers that were programmed according to the individual requirements of the horses. The herd consisted of fifty to sixty privately owned mares and geldings which belonged to different breeds and were between two and twenty nine years in age. The area of observation measures approximately 30 hectares. Data collection took place between June 2018 and Febuary 2019. As horses entered the herd during this time, the number of horses for which permission was given by the owners to participate in the study varied from forty to fifty two. The data was anonymised in a manner, that the horses were numbered randomly and GPS data was only stored associated to the respective number.

The GPS sensors conducted a measurement every 10 s. Coordinated Universal Time (UTC) date, UTC time, local date, local time, validity parameters for the measurement, latitude, longitude, height, as well as speed and distance between two measurements were recorded. All data was stored on the sensor and the battery runtime of the sensors was about 36 h. An accuracy in position measurement of ±1 m was given in the manual. Seven GPS sensors were preliminary used for testing the measurement precision in the field. Two different outdoor locations and testing days were chosen to record the position of motionless sensors for the length of a complete battery runtime. Calculated were the offset between two measurements averaged over all sensors in the test and the averaged offset in positions of two sensors lying directly next to each other.

Two structurally identical sensors (‘a’ and ‘b’) for each horse and two collars were available and were switched every 36 h for the full herd in order to recharge the batteries and to manually transfer the recorded data to a computer. As a result, each GPS data file contained data of approximately 36 h. The horses were neither fixed nor removed from the paddock during the sensor exchange in order to minimize the time the horses were kept from their usual activities. Therefore, the exchange sensors were already recording and taped to the exchange collars prior to being attached to the horse. Thus, the data in the resulting raw GPS files was describing positions of one horse and, additionally, of the person who has been walking through the paddock area to change the sensors (Section 3.1.3).

The example analysis presented in this article used data recorded between 2nd of June 2018 and 3rd of July 2018. In that period forty horses were equipped with a GPS sensor.

### 2.2. Ethical Statement

All horses were normally kept animals. The disturbance of the animals is kept to a minimum as it was the study was designed to fit well into the normal routine of the boarding facility, and it would also have impacted the analysis of herd behaviour otherwise. The authors declare that the “German Animal Welfare Act” (German designation: TierSchG) and the “German Order for the Protection of Animals used for Experimental Purposes and other Scientific Purposes” (German designation: TierSchVersV) were applied. No pain, suffering or injury was inflicted on the animals during the experiment. The animals were used to the collars attached to them, as the boarding facility applies these collars to control the feeding stations. The agreement of the owners of all participating horses was ensured.

### 2.3. Data Preprocessing and Analysis of Contact Networks

The raw GPS data was preprocessed by the self implemented “HerdGPS-Preprocessor” filtering software, which is in detail described in Section 3.1. This resulted in one file per day and horse which holds only GPS data from the horse, as the positions of the sensor changing person have been removed (Section 3.1.3). For the filtering of GPS data, a grid of squares with five meter side length was used (Section 3.1.1). From the information which horse was located in which square of the grid, visualisations of the usage of space were generated.

Furthermore, the software outputs contact lists (Section 3.1.5) from which contact networks could be derived and analysed. Hereby, two horses were to be defined in contact, when their positions were found within the side length of the squares of the grid. Exemplarily, an aggregation window of 60 min was chosen, which led to 768 (=24 h × 32 days) hourly contact networks. For all generated hourly networks density, diameter and the clique structure were calculated using the R package ‘igraph’. The density calculates as the ratio of the edges present in the network to the number of all possible edges. This value describes to which amount animals exploited their opportunities for social contacts. The diameter of a network is the maximal length a shortest path between two nodes can have, i.e., the shortest distance between the two most distant nodes in the network. A clique is a totally connected sub network, that means a group of nodes which are pairwisely connected to each other. As clique metrics the number of cliques with six or more horses as well as the size of the largest clique were analysed.

The hourly networks were plotted with different cliques coloured differently. To analyse dependencies on the daytime, the 768 values for the two clique metrics, density, and diameter, respectively, were grouped both after the hour and after the day, and those groupings were tested for significance using Kruskal-Wallis tests. Afterwards pairwise Wilcoxon tests with Bonferroni corrections were conducted. Boxplots were generated for the clique metrics and the density. As the diameter values were integers from a relatively small range, point plots instead of boxplots were produced.

## 3. Results

### 3.1. Software Tool “HerdGPS-Preprocessor”

**Figure 1:**
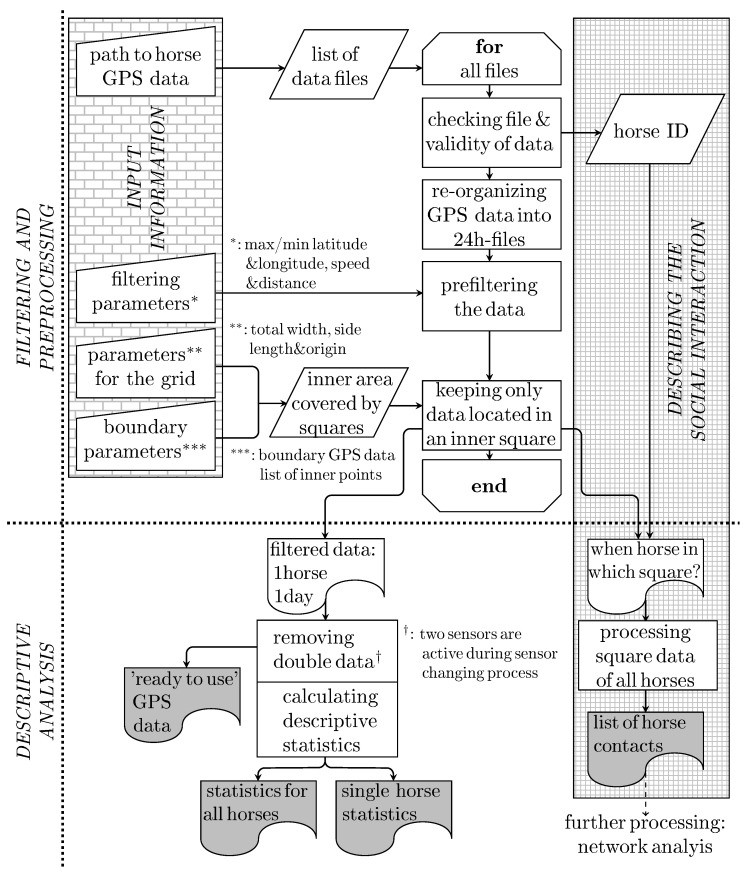
Flow chart of “HerdGPS-Preprocessor”. Above the dotted line the part of the program that prepares and filters the data is illustrated. In the part presented beneath the dotted line the preprocessed data is used for descriptive purposes. The roughly gridded box on the left contains all manually given input information. Each data file—possibly containing GPS data from several days—is processed individually, whereby the held data is reorganized to generate one file per horse and day. For the main filtering a grid is lain over the observation area and only GPS data from inside the grid squares is kept. With this, also the information which horse is when in which square is written to file. The finely gridded box on the right contains all stages towards a full description of the social interactions between the horses. The output documents are marked in grey.

“HerdGPS-Preprocessor” was written in R version 3.5.1 [53] and uses the packages ’stringr’ (version 1.3.1), ’tictoc’ (version 1.0), ’dlm’ (version 1.1-5), and ’futile.logger’ (version 1.4.3). The general process is divided in two parts (*FILTERING AND PREPROCESSING* and *DESCRIPTIVE ANALYSIS*) and visualized in the flow chart in Figure 1. More detailed information regarding the single calculation steps in “HerdGPS-Preprocessor” and the outcomes are given in Section 3.1.2, Section 3.1.3, Section 3.1.4 and Section 3.1.5. “HerdGPS-Preprocessor” can be downloaded [54], and is provided with a manual on how to configure the software to individual user needs. The application of “HerdGPS-Preprocessor” is not limited to horses or farm animals.

#### 3.1.1. Construction of a Grid on the Area of Observation

##### Grid

Starting from a user-given origin and total width, “HerdGPS-Preprocessor” is generating a grid consisting of squares of a predefined side length. Hereby, a small side length increases computational costs (Table 1) but improves filtering accuracy and the precision of contact definition.

**Figure 2:**
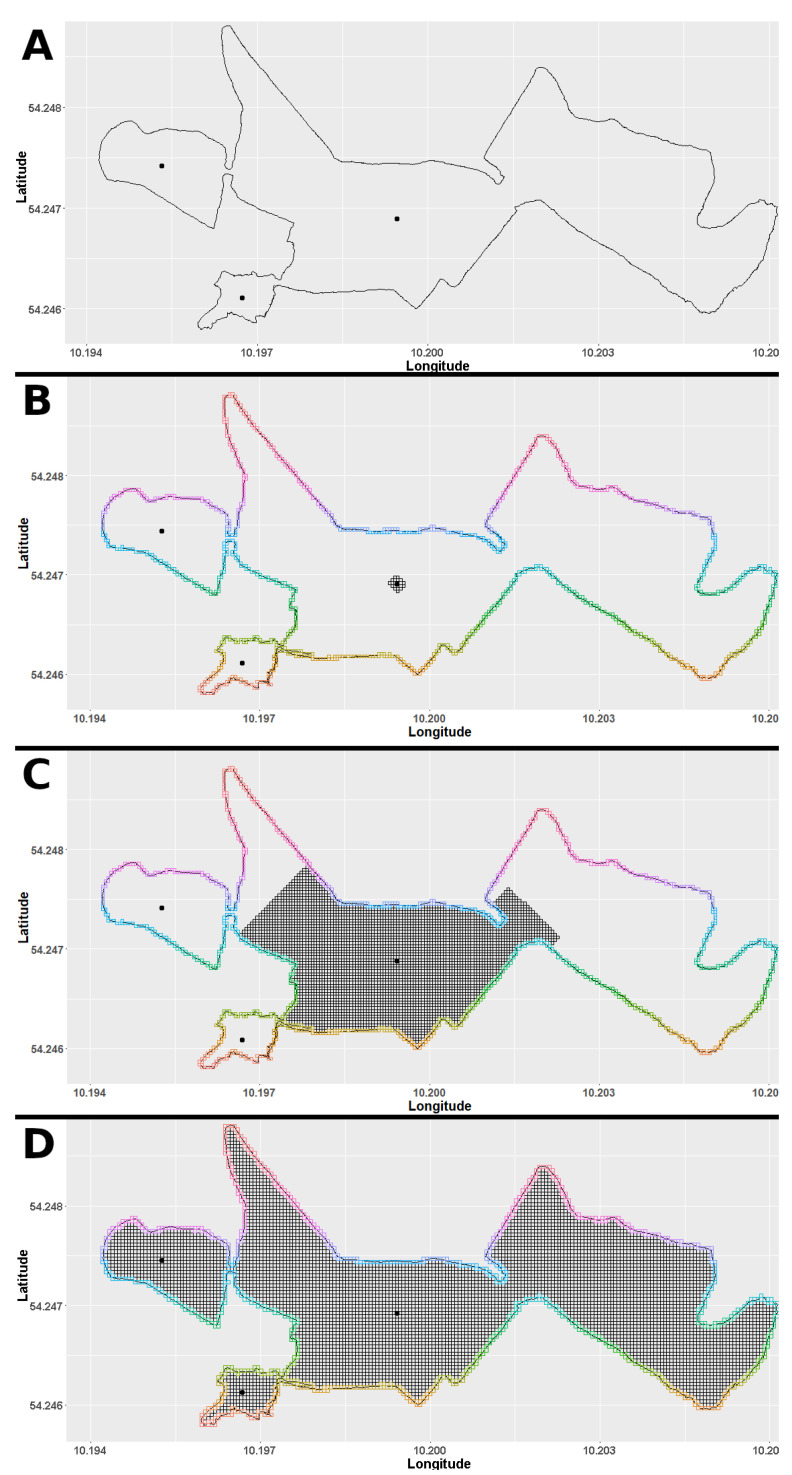
(**A**) Boundary of the area of observation. Black dots mark inner points. (**B**) Starting from an inner point, neighboring squares are added. (**C**) Filling up the area of observation has stopped on the constriction on the bottom left, because it is narrower than the side length. The remaining sectors will be filled starting with the next given inner point. (**D**) Full area gridded with squares of side length 5 m.Gridding.

The grid squares can be addressed by their counts in east and north direction, i.e., square ‘10-3’ is reached by counting ten squares to the east and three squares to the north starting at the origin. The grid is afterwards restricted to the area of observation, e.g., in Figure 2 the area of observation is illustrated covered by squares of side length 5 m. As necessary input for this, GPS data of the boundary of the area of observation has to be provided by the user (Figure 2A). In the case of the underlying study this was achieved by slowly walking along the boundary of the area of observation with a recording GPS sensor. However, surrounding the area with a drone or drawing a polygon along the areas boundary in Google maps also leads to sufficient results. In addition, at least one inner point given in GPS coordinates is needed. Note that, if the area of observation has constrictions, an inner point within each sector should be given (see Figure 2C).

##### Ensuring a Closed Curve of Squares along the Boundary Data

Distances between neighboring GPS measurement on the boundary of the area of observation could get larger than the desired side length. With respect to grid squares, this would lead to an incomplete covering of the boundary, i.e., the series of boundary squares do not form a closed curve. To avoid this, the pairs of GPS measurements with distances larger than the half of the specified side length were determined and the boundary GPS data was linearly interpolated (Table 1).

##### Restricting the Grid to the Area of Observation

In a first step, all squares that contain points of the boundary are specified, and in a second step the inside of the area of observation is filled with squares from the grid. This process starts from the given inner points and is successively checking neighboring squares until a square on the boundary is reached. As can be seen in Figure 2C, the area of observation can have constrictions narrower than the side length. Therefore, it is necessary to specify inner points for all sectors of the area of observation.

#### 3.1.2. Filtering

The filtering of GPS data took place in two steps.

##### Prefiltering

During the prefiltering all GPS measurements were discarded that were located north of the most northern or south of the most southern latitude or west of the most western, respectively, east of the most eastern longitude of the area of observation. After this rough reduction of outliers, all measurements with unnaturally high speed or unnaturally large distances were discarded, in addition. Concerning the application on loose-housing horses (Section 3.3) the thresholds were set to 50 km/h, respectively, 140 m per 10 s. Due to possibly irregular shapes of the area of observation (see Figure 2) erroneous GPS measurements that are located outside the area of observation could still remain in the data set after prefiltering. However, the prefiltering reduces the amount of data run through the computationally more expensive filtering by grid.

##### Filtering by Grid

The main filtering step uses a square grid with user defined side length which was restricted to the area of observation (Section 3.1.1, Figure 2D). For every GPS measurement the square in which it was contained was specified. The measurement was kept only when this square was a square inside the area of observation or a square on the boundary. Thus, all GPS data was bounded to the area of observation with a precision depending on the chosen side length of the grid. The filtered GPS data is reorganised in one file per animal and day and provided by “HerdGPS-Preprocessor” as interim results in csv files. The information about the squares in which each horse was located was stored for the later determination of contacts between horses.

#### 3.1.3. Removing Overlapping Sensor Data

As the two sensors (‘a’ and ‘b’), that were available for each horse, were both recording during sensor exchange in order to minimize the time for this procedure (Section 2.1), overlapping data of these two sensors could be present in the interim results. The files were successively read and cleaned from the sensor data that was not caused by the horse.

**Figure 3:**
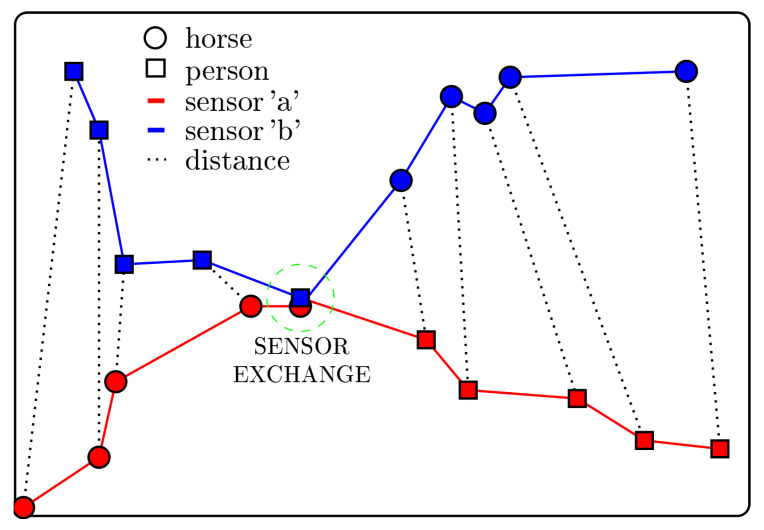
Graphical illustration of recorded GPS data during sensor exchange. The horse is marked by the circles, the person is marked by the rectangles. In the beginning the person is carrying the replacement sensor, i.e., sensor ‘b’ (blue rectangles) and the horse is carrying the sensor ‘a’ (red circles). Within the green dashed circle, the person has exchanged the sensors and is now carrying the replaced sensor ‘a’ (red rectangles). The horse is walking off with sensor ‘b’ (blue circles). For all positions marked with circles or rectangles GPS measurements can be found in the data set. The distances between paired GPS measurements are represented by dotted lines. Those distances reach a minimum with the sensor exchange and increase again afterwards.

For this, as a first step, the time period concerned with overlapping GPS data was determined by specifying the latest data set entry of the replaced sensor, i.e., the sensor attached to the horse that ought to be exchanged, and the first entry of the replacement sensor, i.e., the sensor that is ought to be attached to the horse. Within this period, the sensor changing person has approached the horse with the replacement sensor, exchanged sensors, and has moved away to the next horse with the replaced sensor. It was now necessary to remove the GPS data of the replacement sensor before the exchange and the GPS data of the replaced sensor after the exchange. Thus, as a second step euclidean distances between pairs of GPS measurements of the replacement sensor and the replaced sensor were calculated (Figure 3). The moment of sensor exchange was set to the time, when the distances between the two sensors got smaller than 1 m for the first time. This distance was used due to ±1 m measurement accuracy stated in the manual of the used GPS sensors and can be adapted by the user. In case no existence was given, the first pair exhibiting minimal distance between the sensors was chosen.

Despite the fact that this could be seen as a preprocessing step, it is carried out in part II of “HerdGPS-Preprocessor” (*DESCRIPTIVE ANALYSIS* , Figure 1). This is due to the fact that only the comparison between the data of two sensors within one file makes this data cleaning step possible. This could firstly be achieved after the data from both sensors has been reorganized in part I of “HerdGPS-Preprocessor” (*FILTERING AND PREPROCESSING*, Figure 1). Please note, that this procedure is also working, when 3 or more sensors were used for one animal, as long as only two sensors can be found within twenty four hours. “HerdGPS-Preprocessor” can also be used in a setting with one sensor per animal. The cleaned GPS data was stored in one csv file per animal and day and offered to the user for further analysis.

#### 3.1.4. Calculation of Descriptive Statistics

From the cleaned GPS data descriptive statistics are calculated and provided as two types of csv output files. First, “HerdGPS-Preprocessor” outputs files for single horses containing the number of GPS measurements from the respective day (N), maximal and minimal latitude and longitude, maximum, minimum, mean, median and standard deviation of speed and distance, as well as the total distance. Second, one output file ’STATS_ALL-ANIMALS.csv’ is produced which includes the complete information from the above mentioned files for individual animals in chronological order and an additional column with the animal ID.

#### 3.1.5. Description of the Social Interactions

##### Time Windows

Using the local time associated with each GPS measurement, the day was partitioned into equidistant time windows of multiple lengths: 5 min, 10 min, 15 min, 20 min, 30 min, 1 h, 2 h, 3 h, 4 h, 6 h. For every length the time windows were numbered starting at midnight. For example, 8:34 a.m. has time window number 9 regarding the length 1 h and time window number 52 (=8 × 6 + 4) regarding the length 10 min.

##### Contacts

The information in which squares the horses were located (Section 3.1.2, Filtering by grid) was used to determine whether two animals were positioned within the distance of one side length. This was classified as an undirected contact between those horses. Exemplarily, the line ‘10, 12, 23–41, 6’ would stand for a contact between horses 10 and 12 located by square ‘23–41’ (counted 23 squares eastwards and 41 squares northwards from the origin) during time window 6. E.g., with window length 2 h this would be between 10 a.m. and 12 a.m. See Supplementary Material, Listing S1 for an example.

### 3.2. Test of GPS Sensor Precision

The offset between two measurements averaged over all motionless sensors in the test was 0.018 m. The averaged offset in position between two sensors lying next to each other was 1.78 m in latitude and 0.34 m in longitude.

### 3.3. Application of “HerdGPS-Preprocessor” to GPS Data of Horses

Exemplarily for the area of observation in the present study, Table 1 illustrates the quadratic growth in numbers of squares used for filtering when the predefined side length decreases.

**Figure 4:**
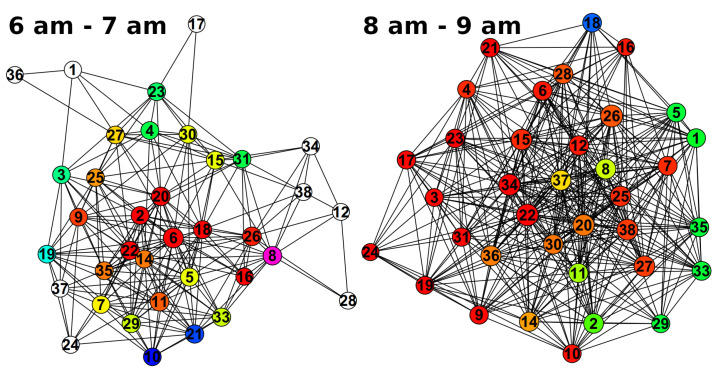
Graph plots of two hourly networks both representing contacts between horses on the 3rd of June 2018. The nodes represent the horses and the edges represent undirected contacts between them within the given hour. Different cliques are coloured differently, whilst nodes belonging to more than one clique are coloured only with regard to the larger clique. White nodes do not belong to any clique.Example networks.

In Figure 4 example networks associated with two hours in the morning of the same day are depicted, illustrating differences in the social structure. Between 6 a.m. and 7 a.m., 50 cliques with more than six horses were observed and the largest clique consisted of eight horses. The network had a diameter of four and a density of 0.34. In the hour from 8 a.m. to 9 a.m. 181 cliques were found, the largest containing twelve horses. Diameter and density of that network were 2 and 0.61, respectively. More examples of hourly contact networks can be found in Figure S1 and Table S1 in the Supplementary Material.

**Figure 5:**
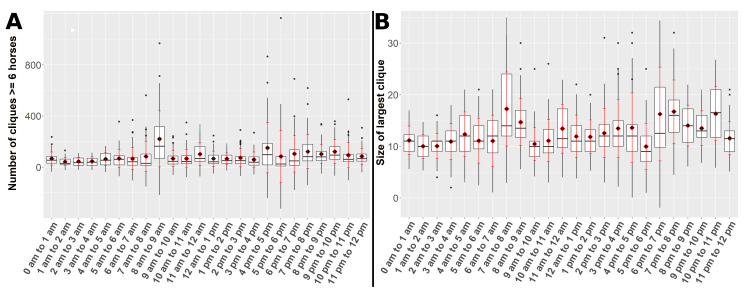
The underlying hourly contact networks were obtained between the 2nd of June and the 3rd of July 2018. The red diamonds mark the means and the red bars represent the mean ± standard deviation. (**A**) Boxplot displaying the numbers of cliques with more than six horses. (**B**) Boxplot displaying the sizes of largest cliques.Boxplots of the clique metrics.

**Figure 6:**
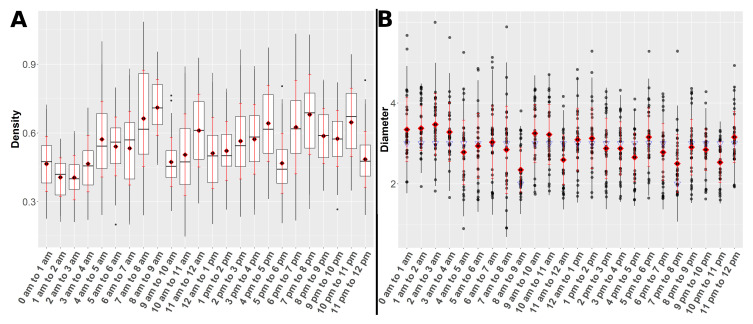
The underlying hourly contact networks were obtained between 2nd of June and 3rd of July 2018. (**A**) Boxplot of the density values. The red diamonds mark the means and the red bars represent the mean ± standard deviation. (**B**) Pointplot of the diameter values. The red diamonds mark the means and the red bars represent the mean ± standard deviation. The blue triangles mark the medians. In order to avoid overplotting of points as the diameter only takes integer values between one and six in this analysis, 1.5% position jitter was allowed.Visual representation of the network parameters.

**Figure 7:**
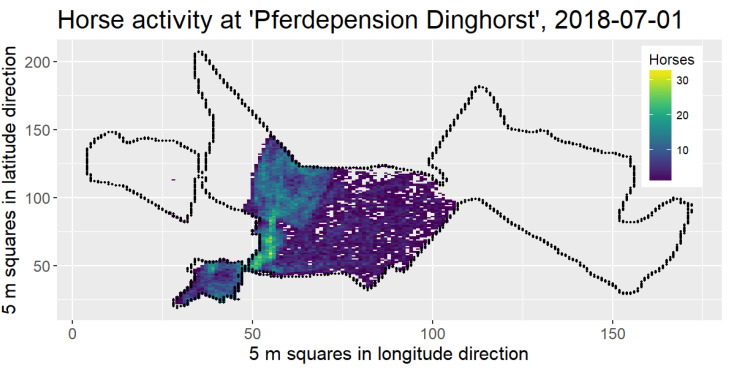
Visual representation of horse activity exemplarily for the 1st of July.

Figure 5 and Figure 6 present the clique metrics and the network parameters density and diameter of all hourly networks derived between the 2nd of June and the 3rd of July 2018, respectively, grouped by hours. The boxplots and the point plot with regard to the grouping after days have been moved to the supplementary material (Figures S2, S3, S5 and S6). In the supplementary material also plots of the courses of the clique metrics, density and diameter could be found (Figures S4, S7, and S8). In Figure 7 an example heatmap of horse activity is illustrated.

Widest interquartile ranges and highest means in the number of cliques with more than six horses could be observed for the hours 8 a.m. to 9 a.m. as well as 4 p.m. to 5 p.m., while this metric behaved relatively stable outside these time periods. The size of the largest clique showed the widest interquartile range and the highest mean in the hour 7 a.m. to 8 a.m. This metric varied stronger over the day compared to the number of cliques and showed wide interquartile ranges and high means between 6 p.m. and 11 p.m.

Means in density showed increasing behaviour towards the hour 8 a.m. to 9 a.m. and a significant drop towards the hour 9 a.m. to 10 a.m. Network densities were increasing again between 11 a.m. and 5 p.m., followed by another significant drop towards the hour 5 p.m. and 6 p.m. Regarding the hours after 6 p.m., the density values remained on a medium to high level until a significant decrease towards the hour 11 p.m. to 12 p.m. Medians in diameter constantly equalled three except from a median equal to two in the hours 8 a.m. to 9 a.m. and 7 p.m. to 8 p.m. The means were also centred around three while the hour 8 a.m. to 9 a.m. mirrored the aforementioned exception. In order to avoid overplotting of points as the diameter only takes integer values between one and six in this analysis, 1.5% position jitter was allowed for this point plot.

The grouping by hours had a significant effect on the clique metrics as well as density and diameter (*p* < 0.05). The grouping by days had a significant effect on the clique metrics but not on density or diameter (*p* < 0.05). The significant pairwise differences found in the corresponding multiple comparisons were moved to the supplementary material (Tables S2–S7).

## 4. Discussion

This article presents a setting for the collection of GPS data from large groups of animals in which both gaps in data recording and interferences with the animals were minimized. The software tool “HerdGPS-Preprocessor” for preprocessing GPS data prepares the raw position data for further statistical analysis and possible static and dynamic network analysis. While presented in terms of a specific study, the suggested software tool is transferable to other experimental setups.

Knowing where each single animal is located in a paddock area or on pasture serves as valuable information in terms of individually walked distances, individual speed performance and the utilisation of space. Furthermore, combining the position information of multiple animals enables the analysis of contacts between animals, i.e., herd structure and social interactions. However, the gathering of reliable coordinates of multiple animals can be challenging. Image processing techniques are one method for the determination of animal position data. Reference [50] successfully developed a framework based on multiple time lapse cameras and machine learning techniques to track flocks of goats on pasture. Nevertheless, in a camera based solution the area of observation has to be registered from the fields of view of several cameras, thus, a transfer to a larger area might come with the need of additional cameras, while the application of GPS sensors automatically generalizes to any area size. Another advantage lies in the geodesic coordinates output by GPS sensors. This makes it unnecessary to transform positions measured in the recording camera’s coordinate system into a joint coordinate system of all cameras [55,56,57]. Furthermore, the positions were determined as the projections of centroids of the found objects onto the ground floor in the image [50] and might depend on how the animal is oriented within the image. Position data extracted this way might, therefore be prone to error when it comes to the exact determination of movement. Whereas, in [51,52] GPS sensors are successfully used to record movement during horse training and games of Polo, respectively. In contrast to recording positions of the animals via GPS or image based methods and afterwards specifying the interactions, proximity loggers are a way to determine the contacts of animals directly, while storing the information when another proximity logger was within a predefined read-range. Using proximity loggers, reference [7] proved that number and duration of bull-cow affiliations significantly increased when cows were in estrous, and reference [6] analysed the social group structure of a herd of 110 dairy cows. However, as the ultra-high frequency waves can be reflected or blocked by objects, for example, other animals, exact distance will vary. The low cost GPS sensor used in this study has a reported accuracy of ±1 m, and preliminary tests of the sensor precision (Section 3.2) revealed that it was justified to consider the measurements within the range given in the manual. In this way, describing animal contacts from GPS position data could be considered similarly accurate than the contacts determined by proximity loggers, though it has to be born in mind, that with GPS data additional calculations need to be made to receive contact information. Generally, a gold standard concerning the position of moving animals to evaluate the position measurements is seldomly available, and would come with the necessity of a second measurement system. As an additional advantage compared to proximity loggers, not only the research dealing with contact networks but also analyses of the paths or the walked distances [19,27] as well as the usage of the provided space as shown in Figure 7 could be based on position data. Field observations [33,58] or inspection of video data [59] are further methods to determine animal contacts and evaluate positions and behaviours. Both data collection methods share independence from sensor failure and sensor precision, however, these methods are time consuming and tedious. Reference [44] have proven that even scan sampling with intervals of 30 or 60 min was not an accurate technique for measuring behaviours compared to continuous observation. In addition, with field observations the results might be affected by the presence of the human observer [60]. GPS data could therefore, be seen as technically superior when it comes to outdoor observations of a large groups of animals [61], and has also increasingly been used in ethological studies [62]. Thereby, GPS data is recorded from feral populations to describe their movements and habitats [24,63], as well as to analyse behavioural patterns in farm animals on pasture [20]. However, monitoring an animal herd using GPS sensors, comes with the burden of limited battery life. The studies reviewed by [63] prolonged the battery runtime as position measurements were only reported in time steps of one to several hours to analyse foraging of wild life herbivores, whilst the analysis of animal behaviour and interactions needs to be based on a much higher sampling rate than the analysis of large range movement pattern. Thus, even with a longer battery life the necessity of changing or recharging the battery remains, when animals are recorded over several weeks or months. In addition, a solution needs to be found to keep the disruption in data recording as short as possible in order to avoid gaps in the tracking of the animals.

The present study suggests a setting to conduct GPS based research in which long periods of continuous data collection from a high number of animals are the aspired goal. Thereby, the application of “HerdGPS-Preprocessor” is not limited to horses, but the use with other herd animals is straightforward. Commercially available GPS collars come with the advantage of attachment gear, high quality sensors and long battery life compared to low price GPS sensors. The GPS HAWK was developed by [19] to provide a less costly alternative to commercial kits. However, the GPS HAWK is priced 500 $ per unit, which sums up to 20,000 $ for a herd of forty animals as in the presented example analysis. Herd sizes exceeding forty animals are not uncommon, and the shoulder harness of the GPS HAWK as well as other pre-existing attachment gear might often not be suitable for the species under analysis. A light weight, low price sensor ranging from 50 Euro to 150 Euro with self constructed attachment might, thus, be the best choice for a lot of GPS based studies. Within this price range, two sensors per animal are affordable to deal with the necessary recharging of sensors. Reference [19] gave an overview on the commercially available GPS kits and also listed the extra costs for software, while their programmable GPS HAWK needs no additional software. The tool “HerdGPS-Preprocessor” presented in this article was implemented using the freely available software R [53] and is applicable to the data recorded by various GPS sensors after configuring the header of the data. The presented study design ensured uninterrupted collection of position data at a high sample rate over several months. For this, the GPS sensors were replaced prior to the end of battery life, and two collars per horse were provided. The sensor exchanges were carried out by quickly attaching a collar with an already running sensor so that the animals were as less as possible disturbed in their daily activities, and gaps in the data could be avoided. By determining when the two sensors approach each other and depart again (Figure 3, Section 3.1.3), “HerdGPS-Preprocessor” specifies which sensor is attached to the horse and deals with the overlapping data. In addition to filtering the GPS data from positions outside the predefined area of observation, GPS data were cleaned by removing all GPS measurements associated to unnaturally high speed or walked distances between two consecutive measurements to avoid large jumps in the tracks of individual animals. The thresholds for speed and distance need to be defined according to the application. In the example analysis presented in this article, knowledge on horse behaviour led to the thresholds 50 km/h for speed and 140 m per 10 s for the distance between two measurements, because horses can not only reach high velocities, but also might perform quick changes in direction during play behaviour or as a flight reaction [64]. “HerdGPS-Preprocessor” reorganises the GPS data into one file per animal and day, in order to enable the researcher to additionally clean the data based on knowledge on individual animals or groups that are more homogeneous in age, size or breed than the whole herd. To further smooth the tracks Kalman filtering [65,66] could be applied individually. This is, however, not carried out within “HerdGPS-Preprocessor”. The goal was to provide the data preprocessed but as raw as possible to the user, in order not to interfere with the further analyses for which the user collected the data, as Kalman filters depend on various parameters and need to be configured carefully with regard to the application [67]. “HerdGPS-Preprocessor” transforms between latitude/longitude degrees and meters to determine a square grid with a given side length. The grid was not only used for the reduction of data to the area of observation, but provides additional scientific approaches. “HerdGPS-Preprocessor” automatically aggregated contacts for time window lengths ranging from five minutes to six hours. These contact lists can be used as edge lists in static or dynamic [41,68] analysis of animal contact networks (Section 2.3, Figure 4). The gridding of GPS data could also serves as a basis to analyse the animals space usage (Figure 7) as a way to address the use of resource and animal preferences [28,29]. Furthermore, the descriptive statistics in the output file ’STATS_ALL-ANIMALS.csv’ (Section 3.1.4) can be used for the visualization of data and a large number of inferential methods. With this, “HerdGPS-Preprocessor” independently of the size of the area of observation, the number of animals to monitor or the animal species delivers versatile output for further analysis.

To demonstrate the usability of the “HerdGPS-Preprocessor”, it was applied to GPS data collected from forty horses in a loose-housing boarding facility over a period of one month, and an analysis of social contacts was chosen as example application. Reference [69] showed that for horses in indoor stables even the possibility of nose to nose contact with conspecifics enhances animal welfare and releases positive emotions, while restricted housing conditions give rise to abnormalities in blood cell count and stereotypic behaviours [70,71], and it is known from [8] that horses’ activity depends on their opportunities to move. As in [33] semiferal horses showed social stability regarding clique structure and individual network positions, it is of great interest to analyse the evolving contact structure of an inhomogeneous herd of domestic horses which were given the opportunity to move freely and fulfil their need to interact with conspecifics [72]. Regarding the complete observation month, number and size of cliques as well as the network parameters density and diameter of the hourly networks differ significantly with the time of the day. Results show that horses had only 40 to 50 % of all possible contacts from 11 p.m. to 4 a.m., compared to significantly higher 60 to 75 % during the rest of the day (Section 3.3, Table S6 in the Supplementary Material). The hours between 11 p.m. to 4 a.m. were expected to be the main resting phase, as in Northern Germany in June this period coincides with the absence of day light, however, this resting phase could very precisely be reproduced by the analysis of hourly contact networks. These findings were backed completely by [26], who showed that the travelled mean distances were smallest between 11 p.m. to 4 a.m.

Apart from circadian rhythms which are common in mammals [73], additional factors were expected to have an impact on the behaviour of the horses and their social interaction. While reference [74] determined that gender, reproductive status and type of enclosure influence the time budget of Przewalski horses, in domestic horses influences due to the management are likely. With this, feeding [10,70], the provision of fresh straw or other enrichment material [75], regrouping [76] or cleaning could be named. Looking at the results, the number of cliques with more than six horses as well as the density showed highest means for the hour 8 a.m. to 9 a.m., and the medians and means of the diameter values grouped by hours reached the minimal values. These findings indicate a comparably dense network with a high number of contacts and that a lot of subgroups of horses initiated during that hour. Furthermore, the density values calculated for the hour 8 a.m. to 9 a.m. differed significantly not only from the night time but also from both directly preceding and following hours in the morning and the rest of the day. For the number of cliques and the diameter values the hour 8 a.m. to 9 a.m. was again significantly different from most remaining hours, stressing the exceptional position of this time of the day and suggesting the influence of an external effect. In the boarding facility under analysis approximately at 8:45 a.m. additional pasture was opened. As the horses expected the pasture to be opened, the herd gathered leading to an increased number of contacts and the forming of cliques.

To conclude on the opportunities given by the output from “HerdGPS-Preprocessor”, it was shown that basic methods of network analysis already make it possible to visualize herd structure as well as temporal differences. A distinction between resting and activity phases became possible using the provided contact information as well as to determine external impacts on the herd. In this example analysis, only parameters concerning the complete network have been used. In [43] centrality parameters associated to single nodes like degree or betweeness [31,36] were calculated for a network of equine facilities with regard to disease spreading. With this, the contact lists could be utilized to compare centrality parameters calculated for individual horses to characteristics like gender or age or with descriptive statistics from the “HerdGPS-Preprocessor” output file ‘STATS_ALL-ANIMALS.csv’. It has also to be born in mind, that both the filtering of GPS data and the contact definition could be fine-tuned using a smaller side length for the squares of which the grid consisted (Section 3.1.1, Section 3.1.2 and Section 3.1.5), although this came with a quadratic rise (Table 1) in computational costs. Last but not least, since some ethological aspects can only be analysed from long time period observations in a meaningful way—for example, the establishment of group structure in horses is known to take up to one year [58,59,77]—GPS based studies with long observation periods might be beneficial. The setting presented here and the software tool “HerdGPS-Preprocessor” for reliable and transparent data filtering and preprocessing are valuable for conducting such studies, especially when a high number of animals is involved.

## 5. Conclusions

A setting for GPS based studies with long periods of continuous data collection and large number of animals under analysis was introduced. The software tool “HerdGPS-Preprocessor” for the preparation of GPS data collected from a herd of animals was presented. The cleaned data organized within files associated with one animal and day enables direct statistical analysis or further specialized filtering. Since lists of contacts aggregated within various time window lengths are also output, static and dynamic network analyses are possible.

In an example analysis the “HerdGPS-Preprocessor” was applied to GPS data of forty privately owned horses collected in a boarding facility over a one month period. In the analysis of hourly contact networks via network parameter density, diameter and metrics associated to the clique structure, a distinction could be made between a nightly resting phase and daily activity. Furthermore, the external effect of pasture opening could be reproduced from the network parameters.

## Figures and Tables

**Table 1:** With decreasing predefined side length in meters (column 1), the numbers of points needed to be filled to obtain solid boundary coordinates (column 3) as well as number of squares covering the boundary (column 5) and the area of observation (column 6) increased quadratically.

Side	Points of org. Boundary	Points of Filled up Boundary	No. Boundary Squares org.	No. Boundary Squares Filled	No. Area Squares
10 m	4641	4652	567	647	3324
6 m	4641	4716	930	1100	8868
5 m	4641	4893	1111	1303	12,633
4 m	4641	5200	1360	1649	19,551
3.5 m	4641	5636	1803	1886	25,420
3 m	4641	6302	1803	2196	34,391
2.5 m	4641	7402	2141	2614	49,233
2 m	4641	8665	2577	3309	76,567
1.5 m	4641	11,734	3242	4402	135,397
1 m	4641	17,049	4108	6672	303,013

## Supplementary Materials

The following are available at https://www.mdpi.com/2076-2615/10/10/1932/s1. Figure S1: Plots of the hourly networks corresponding to the extracted contacts in Listing S1. Networks are based on data from 1st of July 2018. Different cliques are coloured differently, whilst nodes belonging to more than one clique are coloured only with regard to the larger clique. White nodes do not belong to any clique, Figure S2: Boxplot representing the number of cliques with more than six horses grouped by days. Figure S3: Boxplot representing the sizes of the largest clique in the network grouped by days, Figure S4: Number of cliques with more than six horses (red) and size of the largest clique (blue) plotted on a log10 scale for joint presentation, Figure S5: Boxplot of the density values grouped by days, Figure S6: Pointplot of the diameter values grouped by days, The blue triangles mark the medians. A 1.5% jitter was allowed to avoid overplotting of points. Figure S7: Density values of hourly networks, Figure S8: Diameter values of hourly networks, Table S1: Clique metrics, diameter and density of the example networks in Figure S.1, Table S2: *p*-values from multiple comparisons between different hours of the numbers of cliques with more than six horses. Significant differences (*p* < 0.05) are marked blue, Table S3: *p*-values from multiple comparisons between different hours of the sizes of the largest cliques. Significant differences (*p* < 0.05) are marked blue, Table S4: *p*-values from multiple comparisons between different days of the numbers of cliques with more than six horses in hourly networks. Significant differences (*p*< 0.05) are marked blue, Table S5: *p*-values from multiple comparisons between different days of the sizes of the largest cliques in hourly networks. Significant differences (*p* < 0.05) are marked blue, Table S6: *p*-values from multiple comparisons between density values of different hours. Significant differences (*p* < 0.05) are marked blue, Table S7: *p*-values from multiple comparisons between diameter values of different hours. Significant differences (*p* < 0.05) are marked blue. Supplementary 1: “HerdGPS-Preprocessor”—A manual in bullet points.
