# Supplementary material for: “HerdGPS-Preprocessor”—A Tool to Preprocess Herd Animal GPS Data; Applied to Evaluate Contact Structures in Loose-Housing Horses
Source: Animals (Basel). 2020 Oct 21;10(10):1932. doi: 10.3390/ani10101932 (PMC7589659; doi:10.3390/ani10101932)
Supplement: Supplementary file 1 [file animals-10-01932-s001.zip › PreprocessAnimalGPSdata_BulletPointManual.pdf]

“HerdGPS-Preprocessor” – A manual in bullet points

J. Salau*, F. Hildebrandt, I. Czycholl, J. Krieter

*: Corresponding author
Institute of Animal Breeding and Husbandry, Christian-Albrechts-University Kiel, Olshausenstraße 40, 24098 Kiel

After your data has been arranged according to **Preparation of raw data**, open the file ’Config.R’ in a text editor and modify settings of “HerdGPS-Preprocessor” (**Configuration of “HerdGPS-Preprocessor”**). An overview of the files generated by the software is given in **Output of the “HerdGPS-Preprocessor”**. More detailed information could be found in the authors’ main article ’ “HerdGPS-Preprocessor” – A tool to preprocess herd animal GPS data; applied to evaluate contact structures in loose-housing horses ’. Make sure to store the files ’Config.R’, ’Utils.R’ and ’HerdGPS-Preprocessor.R’ in R’s working directory

Preparation of raw data

- GPS data needed to be organized in a directory that contained one subdirectory per animal.
- In these data folders GPS data should be stored in csv files.
- Filenames must consist of
  - the date,
  - the animal identification and
  - a one digit marker (’a’, ’b’), denoting one of two exchange sensors.

  Date and animal identification need to be separated by an underscore, whilst the marker is attached directly to the animal identification, e.g. **’180601_10a.csv’**.

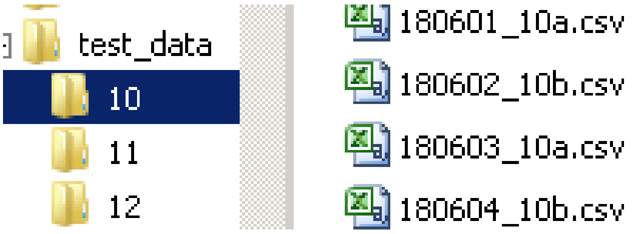

Configuration of “HerdGPS-Preprocessor”

Open the configuration file ’Config.R’ and set the following variables to suit your application.

- Set the path to the directory which contains the data folders (see **Preparation of raw data**).
- Configure variables concerning the used GPS sensor:
  - The header of the data files extracted from the GPS sensor.
  - Formats in which date and time are given (see Listing 1 for an example).

Listing 1: Extract from the configuration file ’Config.R’

```
#path to the folders with animal GPS data
ppath <- file.path("C:", "directory_with_data_folders")
########################################
#SPECIFICS REGARDING THE GPS SENSOR
########################################
# Please copy the header of a representative csv file exported from your GPS sensor
# in the input variable 'header'.
header <- c("ï..INDEX", "RCR", "UTC.DATE", "UTC.TIME", "LOCAL.DATE", "LOCAL.TIME", "MS",
    "VALID", "LATITUDE", "N.S", "LONGITUDE", "E.W", "HEIGHT", "SPEED", "Distance")
# Please specify the formats of local date and local time as recorded by your GPS sensor.
format_date <- "%Y/%m/%d"
format_time <- "%H:%M:%S"
...
```

- Configure variables concerning the area of observation.
  - Specify GPS coordinates of corner points of the minimal rectangle including the area of observation.
  - Enter the name of the file containing GPS data of the boundary of the area of observation (main article, section 3.1.2).
  - GPS coordinates of inner points need to be given as a list (Listing 2).

Listing 2: Extract from the configuration file 'Config.R'

```
####################################
#SPECIFICS REGARDING THE AREA OF OBSERVATION
####################################
# Specify one point respectively for north, south, east, and west to define the minimal
# rectangle including the area of observation, i.e. determine one point each to give the
# most north, most south, most east, and most west coordinates of the area of observation.
end_of_the_world_N <- c(54.248853, 10.196407)
end_of_the_world_S <- c(54.245, 10.196059)
end_of_the_world_E <- c(54.247117, 10.206290)
end_of_the_world_W <- c(54.247284, 10.194165)
#############################
# Provide GPS data of the boundary of the area of observation
name_of_boundary_file <- "walk_around_area.csv"
# Provide list of points within the area of observation
InnerPoints <- list(c(54.246409, 10.197785), c(54.2462, 10.197), c(54.2475, 10.1955))
...
```

- Configure additional variables concerning the prefiltering.
  - Set upper bounds for the covered distance between two consecutive GPS measurements for the boundary (max_dist_boundary) and the GPS data recorded from the animals (max_dist).
  - Define maximal speed for GPS data of the boundary (max_speed_boundary) and GPS data recorded from the animals (max_speed, Listing 3).

Listing 3: Extract from the configuration file 'Config.R'

```
####################################
#SPECIFICS REGARDING (PRE)FILTERING
####################################
# Speed and distance upper bounds to filter the GPS data of the boundary
# (defaults defined for a walking person)
max_dist_boundary <- 20 # m in 10 seconds
max_speed_boundary <- 7 # km/h
# Speed and distance upper bounds for animals (to prefilter the animals GPS data)
max_dist <- 140 # m in 10 seconds
max_speed <- 50 # km/h
...
```

- Configure variables concerning the grid used for filtering.
  - Specify GPS coordinates of a grid origin located south and west of the area of observation.
  - Define a side length in meters.
  - Set a total width in meters large enough that the grid covers the area of observation completely.
  - Specify GPS coordinates of two reference points and the distance between them, to enable a transformation between latitude/longitude degrees and meters (Listing 4, main article, section 3.1.2).

Listing 4: Extract from the configuration file 'Config.R'

```
####################################
#SPECIFICS REGARDING THE GRID
####################################
# Set origin of the grid. Place the origin soutern of the most south and western
# of the most west points of your area of obesrvation to fully have the area of
# observation within the first quadrant.
```

```
origin <- c(54.2455, 10.194)
# Set side length of squares and total grid width. Make sure the total grid width
# is chosen larger than the diameter of the area of observation
side_length <- 5
total_width <- 2000
# For the transformation from latitude/longitude degree to meters, two reference
# points with a known distance between them are needed.
ref_point_1 <- c(54.247030, 10.196699)
ref_point_2 <- c(54.246838, 10.197550)
reference_distance <- 60
...
```

- Set threshold distance for sensor changing between the 'replacement GPS sensor' carried by the sensor changing person and the 'to be replaced GPS sensor' on the animal (main article, section 3.1.4).

  $$\text{sensor\_changing\_distance} \leftarrow 1\,\text{m}$$

- Specify the time window lengths for the aggregation of contacts. Choose from the following:

Listing 5: Extract from the configuration file 'Config.R'

```
# You could save computational time, if you specify the time windows of interest.
time_window_lengths <- c("360min_window", "240min_window", "180min_window", "120min_window",
    "60min_window", "30min_window", "20min_window", "15min_window", "10min_window",
    "5min_window")
...
```

- If you want the “HerdGPS-Preprocessor” to output statistical visualization (see **Output of the “HerdGPS-Preprocessor”**) of your data, set

  $$\text{data\_visualization} \leftarrow \text{TRUE.}$$

- If you already have a list of squares (i.e. a file ListOfCoveringSquares_Sidelength*side-length*.RData) of the given side length *side-length* for your area of observation, set

  $$\text{calculate\_list\_of\_squares} \leftarrow \text{FALSE.}$$

Save and close 'Config.R'.

Output of the “HerdGPS-Preprocessor”

File name parts set in italics do change with day, time, side length time window length and animal.

- Filtered GPS data ⟶ Stored in directory: Day_datafiles_*date*_*time*
  - 'InterimResult_*day*_*side-length*_*animal*.csv'
    Interim results, filtered GPS data, but not cleaned from overlapping data. Files might contain GPS data of both sensors!
  - **'GPSdata_*day*_Sidelength*side-length*_Animal*animal*.csv'**
    Final preprocessing result
- Descriptive statistics ⟶ Stored in directory: Day_datafiles_*date*_*time*/stats
  - 'STATS_*day*_*animal*.csv'
    Contain minimum, maximum, mean, median, standard deviation and sum of the variables latitude, longitude, speed and distance calculated from a single animal and day.
  - 'STATS4ANIMAL_*animal*.csv'
    Contain number of GPS measurements from the respective day, maximal and minimal latitude and longitude, maximum, minimum, mean, median and standard deviation of speed and distance, the percentage of GPS measurements related to a speed slower than 5 km/h, between 5 km/h and 15 km/h, and faster than 15 km/h, as well as the total distance.

- **’STATS__ALL-ANIMALS.csv’**
  Includes the information of all ’STATS4ANIMAL__*animal*.csv’ files.

- Visualization (if configured) ⟶ Stored in directory: Day__datafiles__*date__time*/stats/visualization
  - ’Boxplot-maxspeed.pdf’, ’Boxplot-meanspeed.pdf’
  - ’Boxplot-maxdistance.pdf’, ’Boxplot-meandistance.pdf’, ’Boxplot-sumdistance.pdf’
  - ’Histogram-maxspeed.pdf’, ’Histogram-sumdistance.pdf’
  - ’MeanDailydistances__byAnimals.pdf’, ’MeanDailydistances__byDays.pdf’

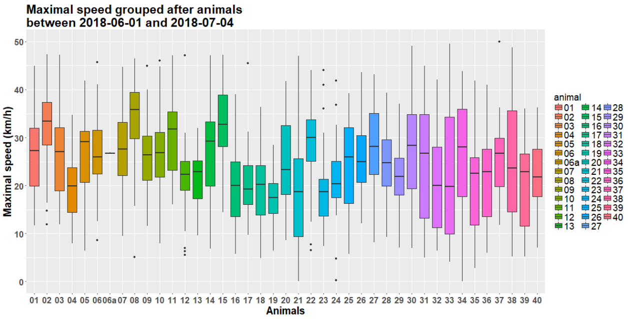

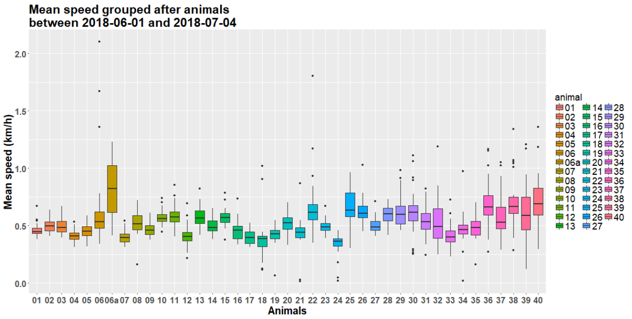

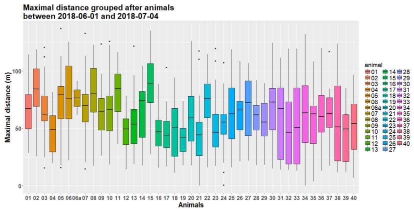

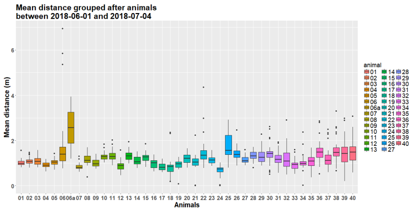

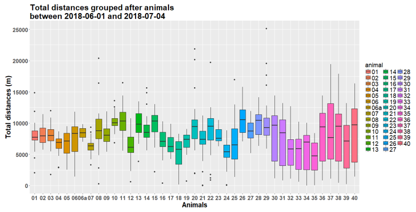

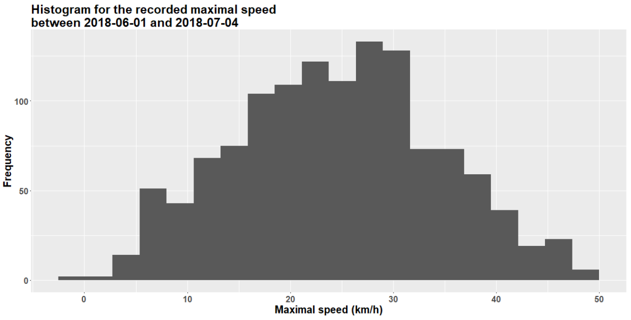

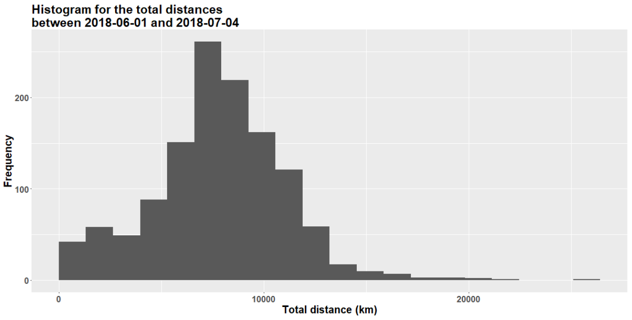

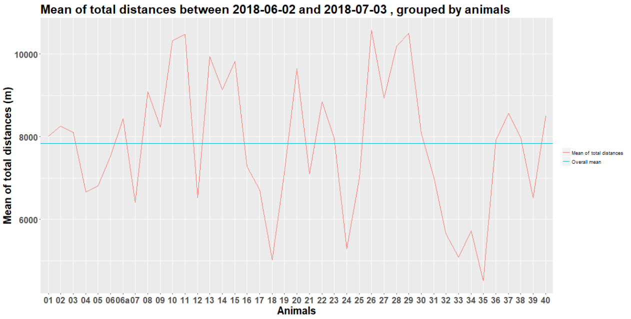

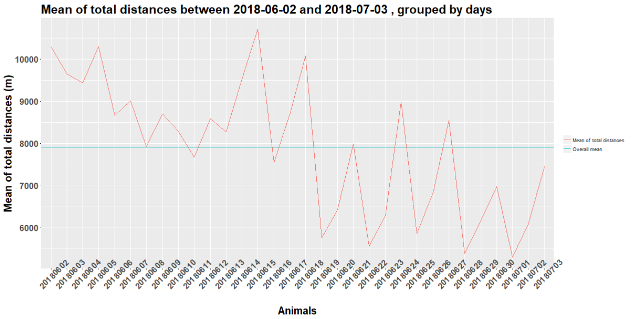

- Square information and contact data ⟶ Stored in directory: Contact__data
  - ’SquareInformation__rawtime__*side-length__day*.csv’
    The information about the squares in which each animal was located at which time.
  - ’SquareInformation__Timewindows__*side-length__day*.csv’
    The local times in ’SquareInformation__rawtime__*side-length__day*.csv’ were transformed to time window numbers.
  - **’ContactNetwork__Timewindow*window-length*min__Sidelength*side-length__day*.csv’**
    Contact lists with square and time window information to be used as edge lists in network analysis.
