# Supplementary material for: “HerdGPS-Preprocessor”—A Tool to Preprocess Herd Animal GPS Data; Applied to Evaluate Contact Structures in Loose-Housing Horses
Source: Animals (Basel). 2020 Oct 21;10(10):1932. doi: 10.3390/ani10101932 (PMC7589659; doi:10.3390/ani10101932)
Supplement: Supplementary file 1 [file animals-10-01932-s001.zip › PreprocessAnimalGPSdata_SupplementaryMaterial.pdf]

Supplementary material to
"HerdGPS-Preprocessor" – A tool to preprocess herd animal GPS data; applied to evaluate contact structures in loose-housing horses

J. Salau*, F. Hildebrandt, I. Czycholl, J. Krieter

*: Corresponding author
Institute of Animal Breeding and Husbandry, Christian-Albrechts-University Kiel, Olshausenstraße 40, 24098 Kiel

S.1 Contact networks

Listing S.1: Extract from the contact list for 1st of July 2018

```
"Animal1","Animal2","Square","TimeWindow(60min)"
"19","33","65-57","5"
"30","37","94-65","5"
"9","10","122-62","5"
...
"8","15","57-52","10"
"23","10","56-52","10"
...
"4","21","127-60","17"
"15","23","88-57","17"
"27","6","95-62","17"
...
"24","8","27-43","22"
"5","17","48-38","22"
```

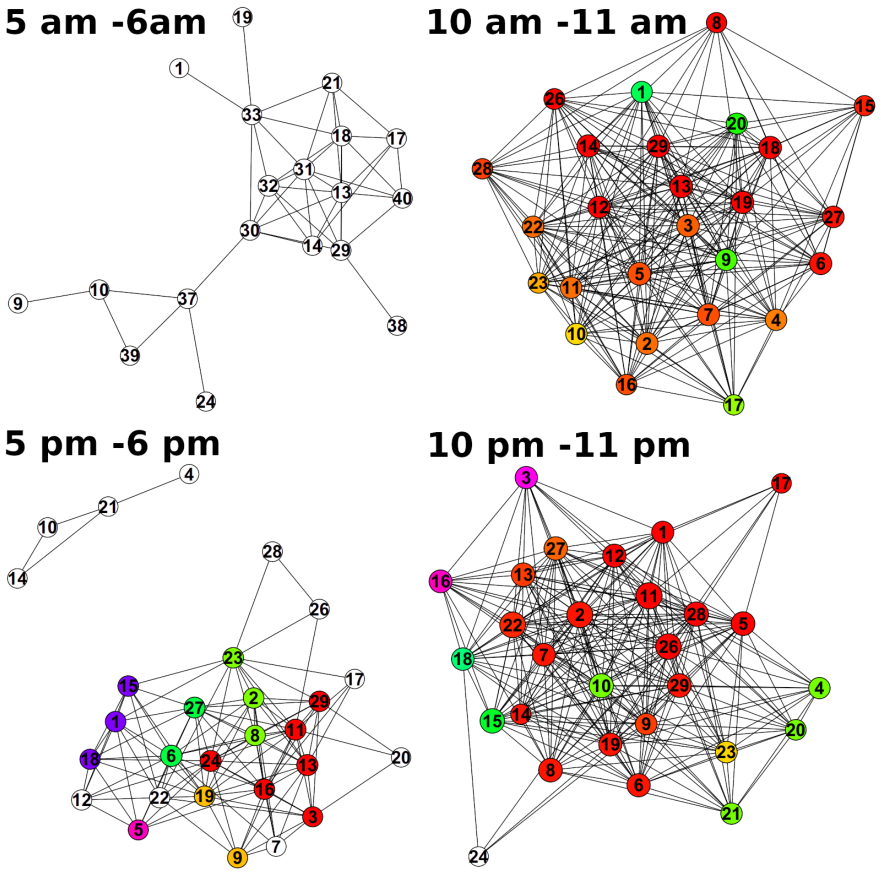

Figure S.1: Plots of the hourly networks corresponding to the extracted contacts in Listing S1. Networks are based on data from 1st of July 2018. Different cliques are coloured differently, whilst nodes belonging to more than one clique are coloured only with regard to the larger clique. White nodes do not belong to any clique.

Table S.1: Clique metrics, diameter and density of the example networks in Figure S.1.

date	hour	cliques >=6	largest_clique	diameter	density
2018-07-01	5 am to 6 am	0	3	5	0.20
2018-07-01	10 am to 11 am	71	11	3	0.65
2018-07-01	5 pm to 6 pm	26	7	3	0.41
2018-07-01	10 pm to 11 pm	27	8	3	0.38

S.2 Clique metrics

In addition to the boxplots presented in Figure 6 in the main article which illustrate the clique metrics grouped by hours, Figures S.2 and S.3 depict the clique metrics grouped by days. The underlying contact networks are based on one hour aggregation windows. The red diamonds mark the means and the red bars represent the mean ± standard deviation.

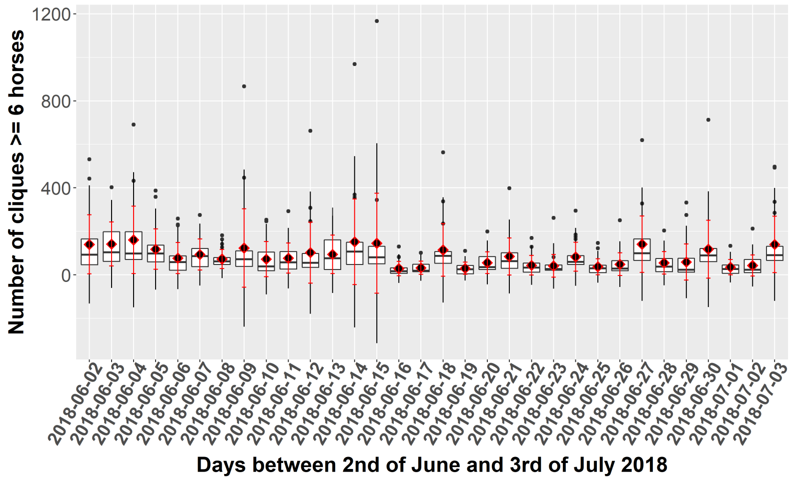

Figure S.2: Boxplot representing the number of cliques with more than six horses grouped by days.

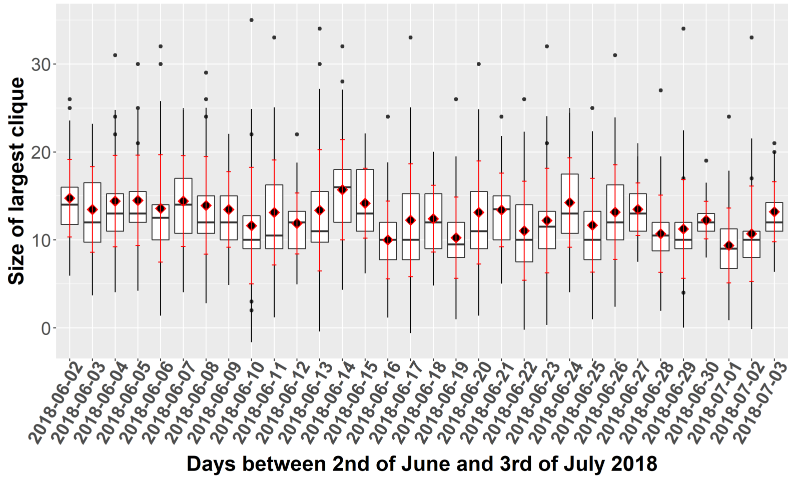

Figure S.3: Boxplot representing the sizes of the largest clique in the network grouped by days.

Figure S.4 presents the course of both clique metrics. As those differ in range, the values were transformed by taking the logarithm of base 10 for the sake of visibility.

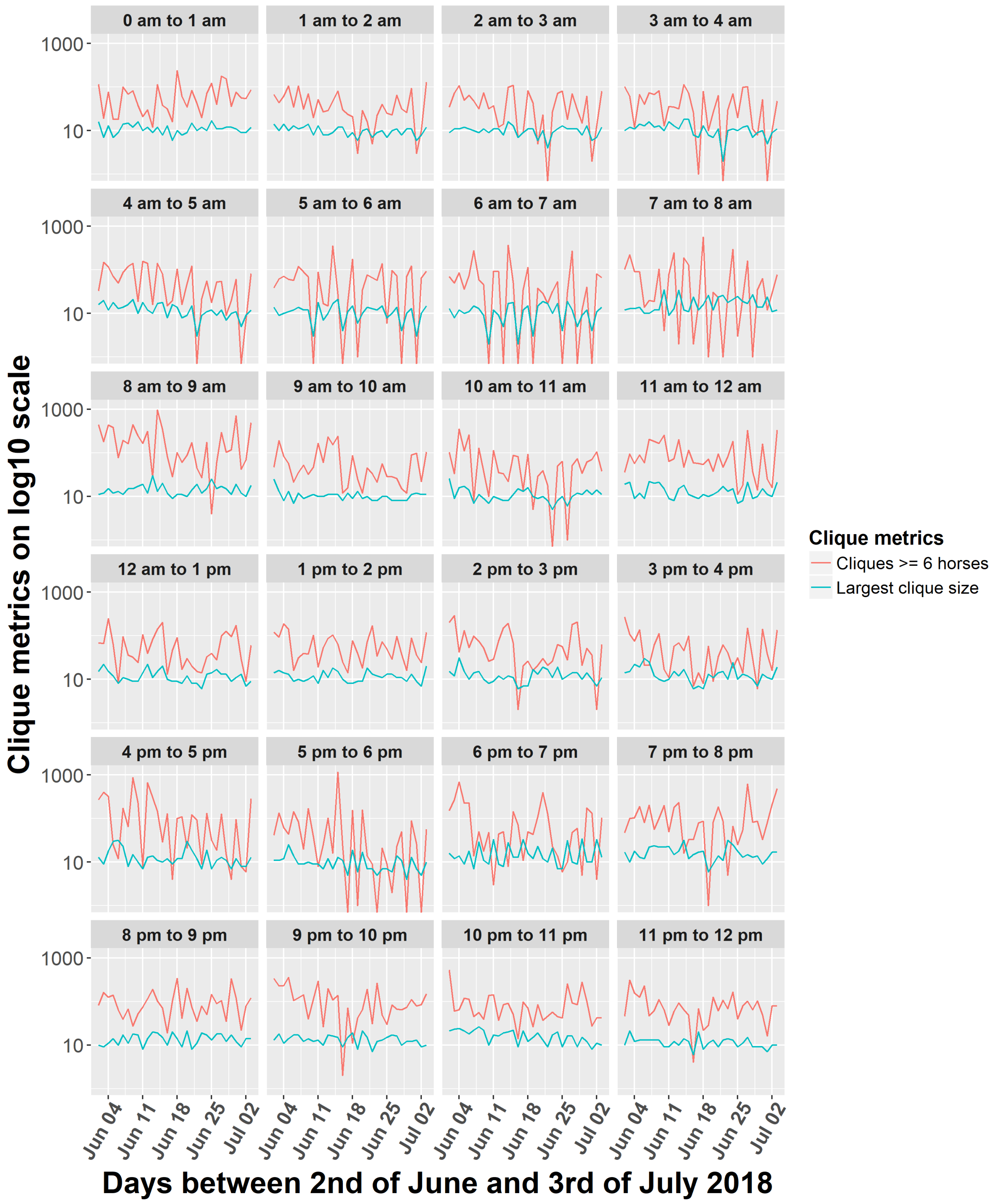

Figure S.4: Number of cliques with more than six horses (red) and size of the largest clique (blue) plotted on a log10 scale for joint presentation.

Tables S.2 and S.3 present p-values from multiple comparisons between different hours of the numbers of cliques with more than six horses, respectively, the sizes of largest cliques. Tables S.4 and S.5 present p-values from multiple comparisons between different days of the clique metrics calculated from the hourly networks. Significant differences ($p < 0.05$) are marked blue. For multiple comparisons a Bonferroni correction was applied.

Table S.2: p-values from multiple comparisons between different hours of the numbers of cliques with more than six horses. Significant differences ($p < 0.05$) are marked blue.

	0am-1 am	1am-2 am	2am-3 am	3am-4 am	4am-5 am	5am-6 am	6am-7 am	7am-8 am	8am-9 am	9am-10am	10am-11am	11am-12am	12am-1pm	1pm-2pm	2pm-3pm	3pm-4pm	4pm-5pm	5pm-6pm	6pm-7pm	7pm-8pm	8pm-9pm	9pm-10pm	10pm-11pm
1am-2 am	1	-	-	-	-	-	-	-	-	-	-	-	-	-	-	-	-	-	-	-	-	-	-
2am-3 am	1	1	-	-	-	-	-	-	-	-	-	-	-	-	-	-	-	-	-	-	-	-	-
3am-4 am	1	1	1	-	-	-	-	-	-	-	-	-	-	-	-	-	-	-	-	-	-	-	-
4am-5 am	1	1	1	1	-	-	-	-	-	-	-	-	-	-	-	-	-	-	-	-	-	-	-
5am-6 am	1	1	1	1	1	-	-	-	-	-	-	-	-	-	-	-	-	-	-	-	-	-	-
6am-7 am	1	1	1	1	1	1	-	-	-	-	-	-	-	-	-	-	-	-	-	-	-	-	-
7am-8 am	1	1	1	1	1	1	1	-	-	-	-	-	-	-	-	-	-	-	-	-	-	-	-
8am-9 am	0.07	0	0	0	0.03	0.09	0.02	0.1	-	-	-	-	-	-	-	-	-	-	-	-	-	-	-
9am-10 am	1	1	1	1	1	1	1	1	0.03	-	-	-	-	-	-	-	-	-	-	-	-	-	-
10am-11 am	1	1	1	1	1	1	1	1	0.06	1	-	-	-	-	-	-	-	-	-	-	-	-	-
11am-12 am	1	0.33	0.88	1	1	1	1	1	1	1	1	-	-	-	-	-	-	-	-	-	-	-	-
12am-1pm	1	1	1	1	1	1	1	1	0.07	1	1	1	-	-	-	-	-	-	-	-	-	-	-
1pm-2pm	1	1	1	1	1	1	1	1	0.08	1	1	1	1	-	-	-	-	-	-	-	-	-	-
2pm-3pm	1	1	1	1	1	1	1	1	0.07	1	1	1	1	1	-	-	-	-	-	-	-	-	-
3pm-4pm	1	1	1	1	1	1	1	1	0.01	1	1	1	1	1	1	-	-	-	-	-	-	-	-
4pm-5pm	1	1	1	1	1	1	1	1	1	1	1	1	1	1	1	1	-	-	-	-	-	-	-
5pm-6pm	1	1	1	1	1	1	1	1	0	1	1	1	1	1	1	1	1	-	-	-	-	-	-
6pm-7pm	1	1	1	1	1	1	1	1	0.9	1	1	1	1	1	1	1	1	1	-	-	-	-	-
7pm-8pm	1	0.08	0.15	0.28	1	1	1	1	1	1	1	1	1	1	1	1	1	0.76	1	-	-	-	-
8pm-9pm	1	0.01	0.02	0.04	1	1	0.67	1	1	1	1	1	1	1	1	0.58	1	0.38	1	1	-	-	-
9pm-10pm	1	0.01	0.03	0.03	1	1	0.59	1	1	1	1	1	1	1	1	0.41	1	0.18	1	1	1	-	-
10pm-11pm	1	0.15	0.74	1	1	1	1	1	1	1	1	1	1	1	1	1	1	1	1	1	1	1	-
11pm-12pm	1	0.26	1	1	1	1	1	1	1	1	1	1	1	1	1	1	1	1	1	1	1	1	1

Table S.3: p-values from multiple comparisons between different hours of the sizes of the largest cliques. Significant differences ($p < 0.05$) are marked blue.

	0am-1 am	1am-2 am	2am-3 am	3am-4 am	4am-5 am	5am-6 am	6am-7am	7am-8 am	8am-9am	9am-10am	10am-11am	11am-12am	12am-1pm	1pm-2pm	2pm-3pm	3pm-4pm	4pm-5pm	5pm-6pm	6pm-7pm	7pm-8pm	8pm-9pm	9pm-10pm	10pm-11pm
1am-2 am	1	-	-	-	-	-	-	-	-	-	-	-	-	-	-	-	-	-	-	-	-	-	-
2am-3 am	1	1	-	-	-	-	-	-	-	-	-	-	-	-	-	-	-	-	-	-	-	-	-
3am-4 am	1	1	1	-	-	-	-	-	-	-	-	-	-	-	-	-	-	-	-	-	-	-	-
4am-5 am	1	1	1	1	-	-	-	-	-	-	-	-	-	-	-	-	-	-	-	-	-	-	-
5am-6 am	1	1	1	1	1	-	-	-	-	-	-	-	-	-	-	-	-	-	-	-	-	-	-
6am-7 am	1	1	1	1	1	1	-	-	-	-	-	-	-	-	-	-	-	-	-	-	-	-	-
7am-8 am	0.02	0	0	0.02	1	0.2	0.4	-	-	-	-	-	-	-	-	-	-	-	-	-	-	-	-
8am-9 am	0.19	0	0	0.19	1	1	1	1	-	-	-	-	-	-	-	-	-	-	-	-	-	-	-
9am-10 am	1	1	1	1	1	1	1	0	0	-	-	-	-	-	-	-	-	-	-	-	-	-	-
10am-11 am	1	1	1	1	1	1	1	0.01	0.06	1	-	-	-	-	-	-	-	-	-	-	-	-	-
11am-12 am	1	1	1	1	1	1	1	1	1	1	1	-	-	-	-	-	-	-	-	-	-	-	-
12am-1pm	1	1	1	1	1	1	1	0.24	1	1	1	1	-	-	-	-	-	-	-	-	-	-	-
1pm-2pm	1	1	1	1	1	1	1	0.2	1	1	1	1	1	-	-	-	-	-	-	-	-	-	-
2pm-3pm	1	1	1	1	1	1	1	1	1	1	1	1	1	1	-	-	-	-	-	-	-	-	-
3pm-4pm	1	1	1	1	1	1	1	1	1	1	1	1	1	1	1	-	-	-	-	-	-	-	-
4pm-5pm	1	1	1	1	1	1	1	1	1	1	1	1	1	1	1	1	-	-	-	-	-	-	-
5pm-6pm	1	1	1	1	1	1	1	0	0.01	1	1	0.76	1	1	1	1	1	-	-	-	-	-	-
6pm-7pm	1	1	1	1	1	1	1	1	1	1	1	1	1	1	1	1	1	0.44	-	-	-	-	-
7pm-8pm	0.01	0	0	0.01	0.82	0.05	0.14	1	1	0	0.01	1	0.12	0.08	0.55	1	1	0	1	-	-	-	-
8pm-9pm	1	0.02	0.04	1	1	1	1	1	1	0.05	0.67	1	1	1	1	1	1	0.04	1	1	-	-	-
9pm-10pm	1	0.01	0.01	1	1	1	1	1	1	0.02	0.84	1	1	1	1	1	1	0.05	1	1	1	-	-
10pm-11pm	0.04	0	0	0.04	1	0.12	0.19	1	1	0	0.03	1	0.27	0.19	0.96	1	1	0	1	1	1	1	-
11pm-12pm	1	1	1	1	1	1	1	0.1	0.7	1	1	1	1	1	1	1	1	1	1	0.03	1	1	0.13

Table S.4: p-values from multiple comparisons between different days of the numbers of cliques with more than six horses in hourly networks. Significant differences (p < 0.05) are marked blue.

	06-02	06-03	06-04	06-05	06-06	06-07	06-08	06-09	06-10	06-11	06-12	06-13	06-14	06-15	06-16	06-17	06-18	06-19	06-20	06-21	06-22	06-23	06-24	06-25	06-26	06-27	06-28	06-29	06-30	07-01	07-02
06-03	1	-	-	-	-	-	-	-	-	-	-	-	-	-	-	-	-	-	-	-	-	-	-	-	-	-	-	-	-	-	-
06-04	1	1	-	-	-	-	-	-	-	-	-	-	-	-	-	-	-	-	-	-	-	-	-	-	-	-	-	-	-	-	-
06-05	1	1	1	-	-	-	-	-	-	-	-	-	-	-	-	-	-	-	-	-	-	-	-	-	-	-	-	-	-	-	-
06-06	1	1	1	1	-	-	-	-	-	-	-	-	-	-	-	-	-	-	-	-	-	-	-	-	-	-	-	-	-	-	-
06-07	1	1	1	1	1	-	-	-	-	-	-	-	-	-	-	-	-	-	-	-	-	-	-	-	-	-	-	-	-	-	-
06-08	1	1	1	1	1	1	-	-	-	-	-	-	-	-	-	-	-	-	-	-	-	-	-	-	-	-	-	-	-	-	-
06-09	1	1	1	1	1	1	1	-	-	-	-	-	-	-	-	-	-	-	-	-	-	-	-	-	-	-	-	-	-	-	-
06-10	1	1	1	1	1	1	1	1	-	-	-	-	-	-	-	-	-	-	-	-	-	-	-	-	-	-	-	-	-	-	-
06-11	1	1	1	1	1	1	1	1	1	-	-	-	-	-	-	-	-	-	-	-	-	-	-	-	-	-	-	-	-	-	-
06-12	1	1	1	1	1	1	1	1	1	1	-	-	-	-	-	-	-	-	-	-	-	-	-	-	-	-	-	-	-	-	-
06-13	1	1	1	1	1	1	1	1	1	1	1	-	-	-	-	-	-	-	-	-	-	-	-	-	-	-	-	-	-	-	-
06-14	1	1	1	1	1	1	1	1	1	1	1	1	-	-	-	-	-	-	-	-	-	-	-	-	-	-	-	-	-	-	-
06-15	1	1	1	1	1	1	1	1	1	1	1	1	1	-	-	-	-	-	-	-	-	-	-	-	-	-	-	-	-	-	-
06-16	0	0	0	0	1	0.03	0.05	0.01	1	1	0.15	1	0.01	0	-	-	-	-	-	-	-	-	-	-	-	-	-	-	-	-	-
06-17	0	0	0	0	1	0.15	0.19	0.03	1	1	0.71	1	0.02	0	1	-	-	-	-	-	-	-	-	-	-	-	-	-	-	-	-
06-18	1	1	1	1	1	1	1	1	1	1	1	1	1	1	0.03	0.06	-	-	-	-	-	-	-	-	-	-	-	-	-	-	-
06-19	0	0	0	0	1	0.13	0.07	0.06	1	1	1	1	0.01	0	1	1	0.03	-	-	-	-	-	-	-	-	-	-	-	-	-	-
06-20	0.54	0.14	0.19	0.79	1	1	1	1	1	1	1	1	1	1	1	1	1	1	-	-	-	-	-	-	-	-	-	-	-	-	-
06-21	1	1	1	1	1	1	1	1	1	1	1	1	1	1	0.19	0.64	1	0.82	1	-	-	-	-	-	-	-	-	-	-	-	-
06-22	0.15	0.02	0.03	0.09	1	1	1	1	1	1	1	1	0.26	0.21	1	1	1	1	1	1	-	-	-	-	-	-	-	-	-	-	-
06-23	0	0	0	0.01	1	0.69	0.52	0.09	1	1	1	1	0.12	0.01	1	1	0.27	1	1	1	1	-	-	-	-	-	-	-	-	-	-
06-24	1	1	1	1	1	1	1	1	1	1	1	1	1	1	0.08	0.28	1	0.1	1	1	1	1	-	-	-	-	-	-	-	-	-
06-25	0.01	0	0	0.01	1	0.64	0.29	0.19	1	1	1	1	0.08	0.02	1	1	0.22	1	1	1	1	1	0.36	-	-	-	-	-	-	-	-
06-26	0.09	0.03	0.03	0.12	1	1	1	1	1	1	1	1	0.48	0.26	1	1	1	1	1	1	1	1	1	1	-	-	-	-	-	-	-
06-27	1	1	1	1	1	1	1	1	1	1	1	1	1	1	0	0	1	0	0.25	1	0.03	0	1	0	0.04	-	-	-	-	-	-
06-28	1	0.19	0.15	1	1	1	1	1	1	1	1	1	1	1	1	1	1	1	1	1	1	1	1	1	1	0.36	-	-	-	-	-
06-29	0.32	0.17	0.16	0.31	1	1	1	1	1	1	1	1	0.52	0.69	1	1	1	1	1	1	1	1	1	1	1	0.19	1	-	-	-	-
06-30	1	1	1	1	1	1	1	1	1	1	1	1	1	1	0	0	1	0	0.37	1	0.06	0	1	0	0.06	1	0.88	0.36	-	-	-
07-01	0.01	0	0	0.01	1	0.37	0.64	0.12	1	1	1	1	0.03	0.02	1	1	0.18	1	1	1	1	1	0.71	1	1	0	1	1	0	-	-
07-02	0.12	0.02	0.02	0.07	1	1	1	0.82	1	1	1	1	0.14	0.16	1	1	0.41	1	1	1	1	1	1	1	1	0.03	1	1	0.08	1	-
07-03	1	1	1	1	1	1	1	1	1	1	1	1	1	1	0	0	1	0	0.19	1	0.03	0	1	0	0.02	1	0.24	0.1	1	0	0.02

Table S.5: p-values from multiple comparisons between different days of the sizes of the largest cliques in hourly networks. Significant differences ($p < 0.05$) are marked blue.

	06-02	06-03	06-04	06-05	06-06	06-07	06-08	06-09	06-10	06-11	06-12	06-13	06-14	06-15	06-16	06-17	06-18	06-19	06-20	06-21	06-22	06-23	06-24	06-25	06-26	06-27	06-28	06-29	06-30	07-01	07-02
06-03	1	-	-	-	-	-	-	-	-	-	-	-	-	-	-	-	-	-	-	-	-	-	-	-	-	-	-	-	-	-	-
06-04	1	1	-	-	-	-	-	-	-	-	-	-	-	-	-	-	-	-	-	-	-	-	-	-	-	-	-	-	-	-	-
06-05	1	1	1	-	-	-	-	-	-	-	-	-	-	-	-	-	-	-	-	-	-	-	-	-	-	-	-	-	-	-	-
06-06	1	1	1	1	-	-	-	-	-	-	-	-	-	-	-	-	-	-	-	-	-	-	-	-	-	-	-	-	-	-	-
06-07	1	1	1	1	1	-	-	-	-	-	-	-	-	-	-	-	-	-	-	-	-	-	-	-	-	-	-	-	-	-	-
06-08	1	1	1	1	1	1	-	-	-	-	-	-	-	-	-	-	-	-	-	-	-	-	-	-	-	-	-	-	-	-	-
06-09	1	1	1	1	1	1	1	-	-	-	-	-	-	-	-	-	-	-	-	-	-	-	-	-	-	-	-	-	-	-	-
06-10	1	1	1	1	1	1	1	1	-	-	-	-	-	-	-	-	-	-	-	-	-	-	-	-	-	-	-	-	-	-	-
06-11	1	1	1	1	1	1	1	1	1	-	-	-	-	-	-	-	-	-	-	-	-	-	-	-	-	-	-	-	-	-	-
06-12	1	1	1	1	1	1	1	1	1	1	-	-	-	-	-	-	-	-	-	-	-	-	-	-	-	-	-	-	-	-	-
06-13	1	1	1	1	1	1	1	1	1	1	1	-	-	-	-	-	-	-	-	-	-	-	-	-	-	-	-	-	-	-	-
06-14	1	1	1	1	1	1	1	1	1	1	1	1	-	-	-	-	-	-	-	-	-	-	-	-	-	-	-	-	-	-	-
06-15	1	1	1	1	1	1	1	1	1	1	1	1	1	-	-	-	-	-	-	-	-	-	-	-	-	-	-	-	-	-	-
06-16	0.08	1	0.44	0.33	1	1	1	1	1	1	1	1	0.08	0.39	-	-	-	-	-	-	-	-	-	-	-	-	-	-	-	-	-
06-17	1	1	1	1	1	1	1	1	1	1	1	1	1	1	1	-	-	-	-	-	-	-	-	-	-	-	-	-	-	-	-
06-18	1	1	1	1	1	1	1	1	1	1	1	1	1	1	1	1	-	-	-	-	-	-	-	-	-	-	-	-	-	-	-
06-19	0.11	1	0.42	0.32	1	1	1	1	1	1	1	1	0.1	0.39	1	1	1	-	-	-	-	-	-	-	-	-	-	-	-	-	-
06-20	1	1	1	1	1	1	1	1	1	1	1	1	1	1	1	1	1	1	-	-	-	-	-	-	-	-	-	-	-	-	-
06-21	1	1	1	1	1	1	1	1	1	1	1	1	1	1	1	1	1	1	1	-	-	-	-	-	-	-	-	-	-	-	-
06-22	1	1	1	1	1	1	1	1	1	1	1	1	1	1	1	1	1	1	1	1	-	-	-	-	-	-	-	-	-	-	-
06-23	1	1	1	1	1	1	1	1	1	1	1	1	1	1	1	1	1	1	1	1	1	-	-	-	-	-	-	-	-	-	-
06-24	1	1	1	1	1	1	1	1	1	1	1	1	1	1	1	1	1	1	1	1	1	1	-	-	-	-	-	-	-	-	-
06-25	1	1	1	1	1	1	1	1	1	1	1	1	1	1	1	1	1	1	1	1	1	1	1	-	-	-	-	-	-	-	-
06-26	1	1	1	1	1	1	1	1	1	1	1	1	1	1	1	1	1	1	1	1	1	1	1	1	-	-	-	-	-	-	-
06-27	1	1	1	1	1	1	1	1	1	1	1	1	1	1	0.33	1	1	0.39	1	1	1	1	1	1	1	-	-	-	-	-	-
06-28	0.17	1	0.93	0.61	1	1	1	1	1	1	1	1	0.17	0.96	1	1	1	1	1	1	1	1	1	1	1	0.68	-	-	-	-	-
06-29	0.33	1	1	1	1	1	1	1	1	1	1	1	0.31	0.73	1	1	1	1	1	1	1	1	1	1	1	0.9	1	-	-	-	-
06-30	1	1	1	1	1	1	1	1	1	1	1	1	1	1	1	1	1	1	1	1	1	1	1	1	1	1	1	1	-	-	-
07-01	0.01	1	0.05	0.03	1	0.22	0.42	0.51	1	1	1	1	0.02	0.06	1	1	1	1	1	0.37	1	1	0.37	1	1	0.04	1	1	0.25	-	-
07-02	0.06	1	0.13	0.18	1	0.98	1	1	1	1	1	1	0.07	0.08	1	1	1	1	1	1	1	1	1	1	1	0.11	1	1	0.66	1	-
07-03	1	1	1	1	1	1	1	1	1	1	1	1	1	1	1	1	1	1	1	1	1	1	1	1	1	1	1	1	1	0.2	0.3

S.3 Density and diameter

In addition to the plots presented in Figure 7 in the main article which illustrate density and diameter grouped by hours, Figures S.5 and S.6 depict density and diameter grouped by days. The underlying contact networks are based on one hour aggregation windows. The red diamonds mark the means and the red bars represent the mean ± standard deviation.

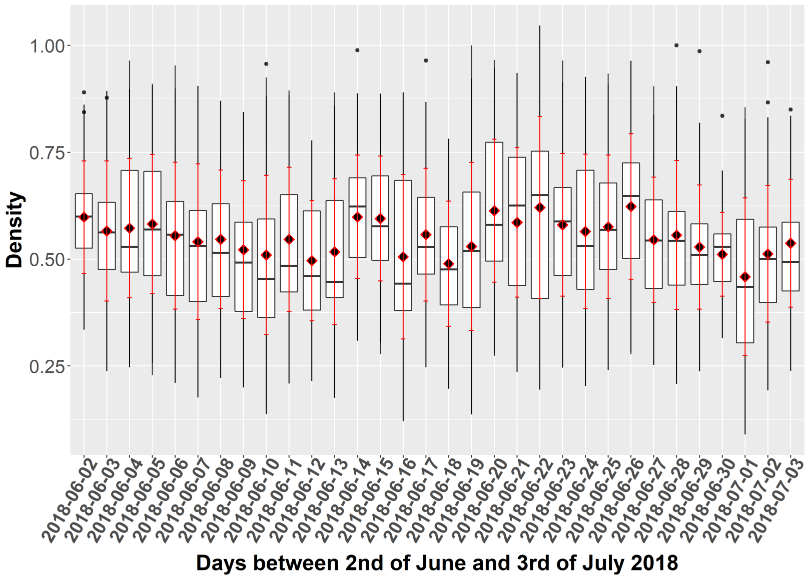

Figure S.5: Boxplot of the density values grouped by days.

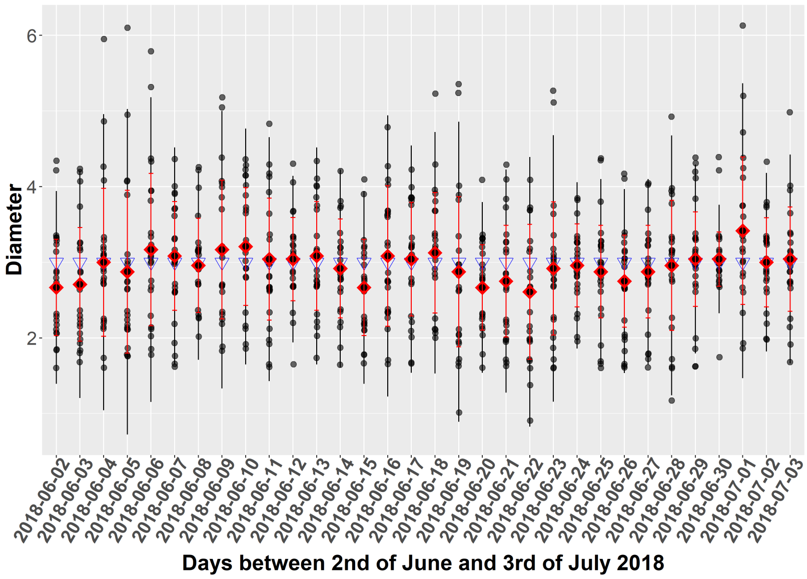

Figure S.6: Pointplot of the diameter values grouped by days. The blue triangles mark the medians. A 1.5% jitter was allowed to avoid overplotting of points.

Figures S.7 and S.8 presents the course of density and diameter of the hourly networks, respectively. Tables S.6 and S.7 present p-values from multiple comparisons between different hours of density, respectively, diameter values. Significant differences ($p < 0.05$) are marked blue. For multiple comparisons a Bonferroni correction was applied.

Grouping after days had no significant effect on the density or diameter of the hourly networks.

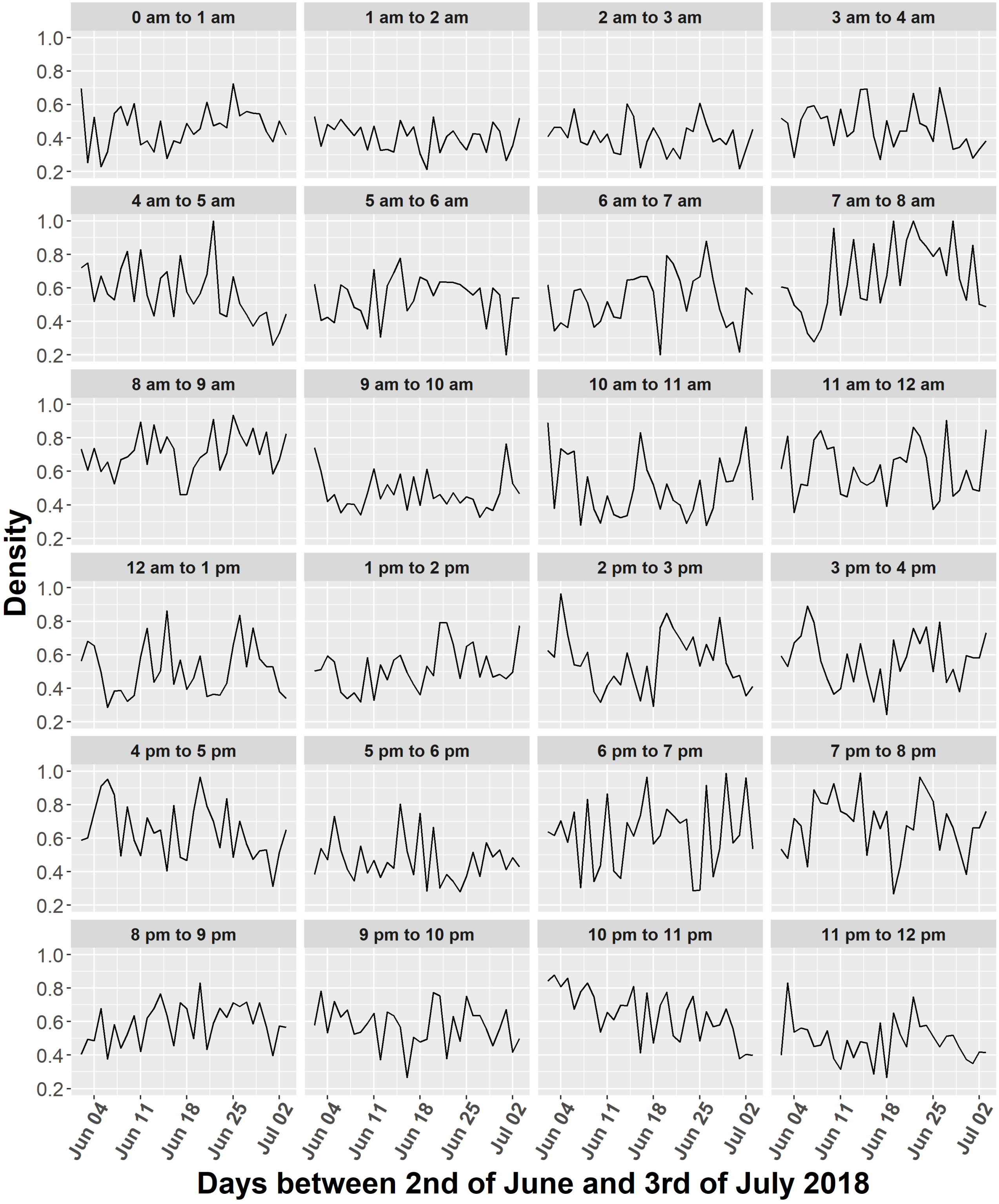

Figure S.7: Density values of hourly networks.

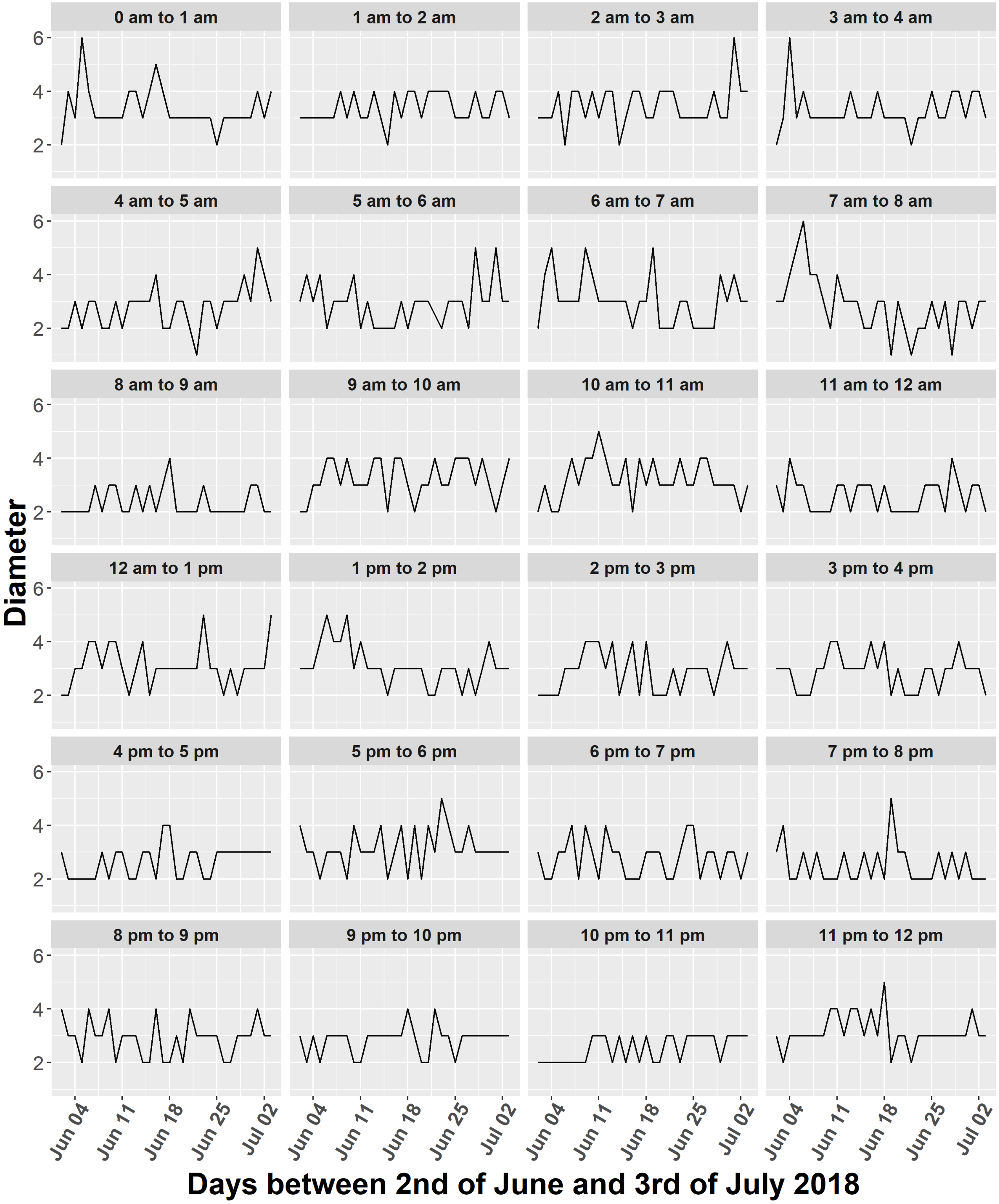

Figure S.8: Diameter values of hourly networks.

Table S.6: p-values from multiple comparisons between density values of different hours. Significant differences ($p < 0.05$) are marked blue.

	0am-1am	1am-2am	2am-3am	3am-4am	4am-5am	5am-6am	6am-7am	7am-8am	8am-9am	9am-10am	10am-11am	11am-12am	12am-1pm	1pm-2pm	2pm-3pm	3pm-4pm	4pm-5pm	5pm-6pm	6pm-7pm	7pm-8pm	8pm-9pm	9pm-10pm	10pm-11pm
1am-2 am	1	-	-	-	-	-	-	-	-	-	-	-	-	-	-	-	-	-	-	-	-	-	-
2am-3 am	1	1	-	-	-	-	-	-	-	-	-	-	-	-	-	-	-	-	-	-	-	-	-
3am-4 am	1	1	1	-	-	-	-	-	-	-	-	-	-	-	-	-	-	-	-	-	-	-	-
4am-5 am	1	0.01	0.01	1	-	-	-	-	-	-	-	-	-	-	-	-	-	-	-	-	-	-	-
5am-6 am	1	0	0.01	1	1	-	-	-	-	-	-	-	-	-	-	-	-	-	-	-	-	-	-
6am-7 am	1	0.18	0.22	1	1	1	-	-	-	-	-	-	-	-	-	-	-	-	-	-	-	-	-
7am-8 am	0.04	0	0	0.05	1	1	1	-	-	-	-	-	-	-	-	-	-	-	-	-	-	-	-
8am-9 am	0	0	0	0	0.09	0	0	1	-	-	-	-	-	-	-	-	-	-	-	-	-	-	-
9am-10 am	1	1	1	1	1	1	1	0.02	0	-	-	-	-	-	-	-	-	-	-	-	-	-	-
10am-11 am	1	1	1	1	1	1	1	1	0	1	-	-	-	-	-	-	-	-	-	-	-	-	-
11am-12 am	0.15	0	0	0.14	1	1	1	1	1	0.04	1	-	-	-	-	-	-	-	-	-	-	-	-
12am-1pm	1	1	1	1	1	1	1	1	0	1	1	1	-	-	-	-	-	-	-	-	-	-	-
1pm-2pm	1	0.03	0.05	1	1	1	1	1	0	1	1	1	1	-	-	-	-	-	-	-	-	-	-
2pm-3pm	1	0.01	0.01	1	1	1	1	1	0.07	1	1	1	1	1	-	-	-	-	-	-	-	-	-
3pm-4pm	1	0	0	1	1	1	1	1	0.08	1	1	1	1	1	1	-	-	-	-	-	-	-	-
4pm-5pm	0.01	0	0	0.01	1	1	1	1	1	0	0.73	1	0.88	0.81	1	1	-	-	-	-	-	-	-
5pm-6pm	1	1	1	1	1	1	1	0.03	0	1	1	0.12	1	1	1	0.96	0	-	-	-	-	-	-
6pm-7pm	0.14	0	0	0.21	1	1	1	1	1	0.56	1	1	1	1	1	1	1	0.29	-	-	-	-	-
7pm-8pm	0	0	0	0	1	0.14	0.1	1	1	0	0.1	1	0.06	0.08	1	1	1	0	1	-	-	-	-
8pm-9pm	0.08	0	0	0.14	1	1	1	1	0.05	0.06	1	1	1	1	1	1	1	0.06	1	1	-	-	-
9pm-10pm	0.15	0	0	0.34	1	1	1	1	0.03	0.06	1	1	1	1	1	1	1	0.15	1	1	1	-	-
10pm-11pm	0	0	0	0	1	1	1	1	1	0	0.3	1	0.25	0.26	1	1	1	0	1	1	1	1	-
11pm-12pm	1	1	1	1	1	1	1	0.11	0	1	1	0.5	1	1	1	1	0.02	1	0.61	0	0.32	0.76	0.01

Table S.7: p-values from multiple comparisons between diameter values of different hours. Significant differences ($p < 0.05$) are marked blue.

	0am-1 am	1am-2 am	2am-3 am	3am-4 am	4am-5 am	5am-6 am	6am-7 am	7am-8am	8am-9am	9am-10am	10am-11am	11am-12am	12am-1pm	1pm-2pm	2pm-3pm	3pm-4pm	4pm-5pm	5pm-6pm	6pm-7pm	7pm-8pm	8pm-9pm	9pm-10pm	10pm-11pm
1am-2 am	1	-	-	-	-	-	-	-	-	-	-	-	-	-	-	-	-	-	-	-	-	-	-
2am-3 am	1	1	-	-	-	-	-	-	-	-	-	-	-	-	-	-	-	-	-	-	-	-	-
3am-4 am	1	1	1	-	-	-	-	-	-	-	-	-	-	-	-	-	-	-	-	-	-	-	-
4am-5 am	1	0.19	0.15	1	-	-	-	-	-	-	-	-	-	-	-	-	-	-	-	-	-	-	-
5am-6 am	1	1	1	1	1	-	-	-	-	-	-	-	-	-	-	-	-	-	-	-	-	-	-
6am-7 am	1	1	1	1	1	1	-	-	-	-	-	-	-	-	-	-	-	-	-	-	-	-	-
7am-8 am	1	1	0.99	1	1	1	1	-	-	-	-	-	-	-	-	-	-	-	-	-	-	-	-
8am-9 am	0	0	0	0	1	0.34	0.16	1	-	-	-	-	-	-	-	-	-	-	-	-	-	-	-
9am-10 am	1	1	1	1	1	1	1	1	0	-	-	-	-	-	-	-	-	-	-	-	-	-	-
10am-11 am	1	1	1	1	1	1	1	1	0	1	-	-	-	-	-	-	-	-	-	-	-	-	-
11am-12 am	0.02	0	0	0.04	1	1	1	1	1	0.12	0.26	-	-	-	-	-	-	-	-	-	-	-	-
12am-1pm	1	1	1	1	1	1	1	1	0.01	1	1	1	-	-	-	-	-	-	-	-	-	-	-
1pm-2pm	1	1	1	1	1	1	1	1	0	1	1	1	1	-	-	-	-	-	-	-	-	-	-
2pm-3pm	1	1	1	1	1	1	1	1	0.81	1	1	1	1	1	-	-	-	-	-	-	-	-	-
3pm-4pm	1	0.74	0.51	1	1	1	1	1	0.27	1	1	1	1	1	1	-	-	-	-	-	-	-	-
4pm-5pm	0.07	0.01	0.01	0.11	1	1	1	1	1	0.31	0.68	1	1	1	1	1	-	-	-	-	-	-	-
5pm-6pm	1	1	1	1	1	1	1	1	0	1	1	0.54	1	1	1	1	1	-	-	-	-	-	-
6pm-7pm	1	0.22	0.17	1	1	1	1	1	1	1	1	1	1	1	1	1	1	1	-	-	-	-	-
7pm-8pm	0	0	0	0	1	1	1	1	1	0.02	0.04	1	0.26	0.11	1	1	1	0.07	1	-	-	-	-
8pm-9pm	1	1	1	1	1	1	1	1	0.22	1	1	1	1	1	1	1	1	1	1	1	-	-	-
9pm-10pm	1	0.09	0.08	1	1	1	1	1	0.09	1	1	1	1	1	1	1	1	1	1	1	1	-	-
10pm-11pm	0	0	0	0	1	1	1	1	1	0.02	0.05	1	0.58	0.23	1	1	1	0.11	1	1	1	1	-
11pm-12pm	1	1	1	1	1	1	1	1	0	1	1	0.2	1	1	1	1	0.62	1	1	0.02	1	1	0.03
